# What is a species? A new universal method to measure differentiation and assess the taxonomic rank of allopatric populations, using continuous variables

**DOI:** 10.3897/zookeys.757.10965

**Published:** 2018-05-10

**Authors:** Thomas M. Donegan

**Affiliations:** 1 Unaffiliated, London, UK

**Keywords:** diagnosis, species limits, species scoring, statistics, subspecies limits, taxonomy

## Abstract

Existing models for assigning species, subspecies, or no taxonomic rank to populations which are geographically separated from one another were analyzed. This was done by subjecting over 3,000 pairwise comparisons of vocal or biometric data based on birds to a variety of statistical tests that have been proposed as measures of differentiation. One current model which aims to test diagnosability (Isler et al. 1998) is highly conservative, applying a hard cut-off, which excludes from consideration differentiation below diagnosis. It also includes non-overlap as a requirement, a measure which penalizes increases to sample size. The “species scoring” model of Tobias et al. (2010) involves less drastic cut-offs, but unlike Isler et al. (1998), does not control adequately for sample size and attributes scores in many cases to differentiation which is not statistically significant. Four different models of assessing effect sizes were analyzed: using both pooled and unpooled standard deviations and controlling for sample size using *t*-distributions or omitting to do so. Pooled standard deviations produced more conservative effect sizes when uncontrolled for sample size but less conservative effect sizes when so controlled. Pooled models require assumptions to be made that are typically elusive or unsupported for taxonomic studies. Modifications to improving these frameworks are proposed, including: (i) introducing statistical significance as a gateway to attributing any weighting to findings of differentiation; (ii) abandoning non-overlap as a test; (iii) recalibrating Tobias et al. (2010) scores based on effect sizes controlled for sample size using *t*-distributions. A new universal method is proposed for measuring differentiation in taxonomy using continuous variables and a formula is proposed for ranking allopatric populations. This is based first on calculating effect sizes using unpooled standard deviations, controlled for sample size using *t*-distributions, for a series of different variables. All non-significant results are excluded by scoring them as zero. Distance between any two populations is calculated using Euclidian summation of non-zeroed effect size scores. If the score of an allopatric pair exceeds that of a related sympatric pair, then the allopatric population can be ranked as species and, if not, then at most subspecies rank should be assigned. A spreadsheet has been programmed and is being made available which allows this and other tests of differentiation and rank studied in this paper to be rapidly analyzed.

## Introduction

This paper aims to help address the “allopatric problem” when determining species rank in taxonomic science. Humans have categorized populations into named groups since the dawn of known civilization (Aristotle c. 350 B.C.) and these were first referred to as “species” over 300 years ago (Willughby 1676, 1678). As defined by Ray (1686): “no matter what variations occur in the individuals or the species, if they spring from the seed of one and the same plant, they are accidental variations and not such as to distinguish a species... Animals likewise that differ specifically preserve their distinct species permanently; one species never springs from the seed of another nor vice versa.”

Sympatric species, which occur together in the same place during the breeding season but do not successfully interbreed to any material extent, are demonstrably real. With enough data and persistence, it is usually possible to determine whether or not sympatric populations interbreed regularly and whether they produce fertile offspring (Mayr 1940) and therefore whether or not the two populations are reproductively isolated. Where hybridization is rare or occurs in narrow zones, this can cause difficulties in delimiting species and may need judgment to be applied.

A traditionally more difficult problem, and the focus of this paper, is that of “allopatric” (Mayr 1942) populations (referred to as “asympatric” by Poulton 1904, 1908, who originally identified this problem), i.e., those which do not occur together in the same geographical place during the breeding season. Allopatric populations can be recognized either as subspecies of polytypic species or as monotypic species under Mayr (1940, 1942)’s scheme. However, allopatric populations should only be ranked as species where they are as distinctive as sympatric species (Helbig et al. 2002). This is not an artificial test. Over a period of time, two disjunct populations facing different selection pressures may differentiate from one another, and at some point, they will attain sufficient differentiation that this can be observed to attain or exceed that shown between sympatric species. At such a point, but not otherwise, it is reasonable to assume that they have speciated.

The subjectivity involved in comparing allopatric species and the rise of molecular science have doubtless encouraged the development of a multitude of different species criteria or concepts. As noted by De Queiroz (1998, 1999), many of these are simply different ways of finding out what a species is, as opposed to being based on different ideas of what species are. However, proponents of these concepts challenge the “comparative approach” to assessing the rank of allopatric populations (e.g., Halley et al. 2017). Under phylogenetic and related species concepts (PSC), diagnosability and monophyly (“clusters of individuals with a pattern of ancestry and descent”: Cracraft 1983) are the hallmarks of species rank. Such “clusters” can be ascertained using molecular biology, a discipline that does not need to be informed by real-world differentiation in morphology, animal sounds, or biometrics. Because all diagnosable units under this model are called species, some PSC proponents have argued for the subspecies rank to be abandoned (Zink 2003). However, whilst molecular research has revolutionized higher-level taxonomy, it is less useful at addressing questions of species rank, since sympatric species show variable intraspecific DNA differentiation, ranging from between 0% to at least 8% (Sorenson et al. 2003, Marks et al. 2002). Many modern ornithological taxonomists seek to take into account the results of both molecular and traditional analyses where possible in assessing rank. Biological species concepts, often integrating “lineage”-based concept thinking (De Queiroz 1998, 1999) remain in prevailing usage among leading checklist committees (e.g., AOU 1998, Helbig et al. 2002, Remsen et al. 2018) and in taxonomic reference works (e.g., Dickinson and Christidis 2014), albeit often informed by molecular data and diagnosability (Sangster 2014).

Whilst statistical and mathematical techniques to analyze molecular data have been a rich field for methodological advancement, the same cannot be said for the study of real world variables. Supportable statistical schemes for assessing between-population differentiation are noteworthy principally by their absence. Those schemes which have been proposed are either widely criticized, only applicable to particular taxonomic groups or vague.

Helbig et al. (2002) developed a set of guidelines for taxonomic committees to assess species and subspecies rank, in the context of de Queiroz (1998, 1999)’s lineage concept. In relation to allopatric populations, these authors recommended that: “The likelihood that allopatric taxa will remain distinct can only be judged by the degree of their divergence, preferably in comparison with taxa that are closely related to the group under investigation and that are known to coexist in sympatry”. They recommended that, in order to be ranked as species, allopatric populations should usually be diagnosable by several discrete or continuously varying characters related to different functional contexts, e.g., structural features (often related to foraging strategy), plumage colors, vocalizations (both often related to mate recognition) or DNA sequences, and the sum of the character differences should correspond to or exceed the level of divergence seen in related species that coexist in sympatry.

This paper will concentrate on the traditional currency of taxonomy: continuous variables such as those based on measurement of specimens, whether in the museum or in the field. Many researchers and advanced amateurs do not have a molecular laboratory available and few genera have been exhaustively sampled in a way that includes multiple individuals at population level. In contrast, vocal and biometric data are easy to collate, accessible to many and cheaper to analyze. A wide variety of other ‘real world’ organism characters are capable of measurement as continuous variables. For vocalizations, lengths or acoustic frequencies of notes can be measured using sonograms, for example. Coloration can be measured using spectrometry. Non-continuous or discrete variables, e.g., presence or absence of a particular character and molecular markers, can be analyzed best using cladistics and other phylogenetic tools and are not covered here in detail.

Hubbs and Perlmutter (1942) proposed that, in order to assess diagnosability using continuous variables, taxonomists should calculate the distance between the means of the two populations for a particular character and measure that distance in terms of standard deviations (SDs), a measure referred to in statistics as “effect size”. Where the means of two populations differ by four average SDs, then under a normal distribution with infinite sample size, there is no overlap between data to 95% confidence and the populations can be considered “diagnosable” for the character in question. As noted by McKitrick and Zink (1988) and Remsen (2010), aiming for 100% diagnosability is conceptually and methodologically unreasonable. 95% is the standard confidence internal in science, the benchmark for assessing diagnosability using discrete characters (Wiens and Servedio 2000, Walsh 2000) and the benchmark for testing diagnosis using continuous variables (Isler et al. 1998). Hubbs and Perlmutter (1942) further proposed a “50% diagnosis” test that might be used for assessing subspecies rank, where populations differ by two SDs: effectively denoting differentiation of a character half-way towards diagnosability. Later, a 75%/99+% diagnosis test for subspecies (e.g., Amadon 1949, Patten and Unitt 2002) was developed and became more widely used. It has more recently been proposed that full (95% statistical) diagnosability in a single character should be the benchmark for subspecies, which is synonymous with a PSC species definition (Remsen 2010).

Isler et al. (1998) modified Hubbs and Perlmutter (1942)’s tests by taking into account sample sizes using student *t*-distribution values rather than bare SDs, to measure the difference between population means (detailed below under Methods: Level 5). This resulted in a model for measuring differentiation and assessing species rank that effectively requires an elevated distance between means of two populations, with greater distances for data using smaller sample sizes. Based on studies of closely related sympatric birds in a particular bird family, the antbirds (Thamnophilidae), Isler et al. (1998) concluded that three diagnostic vocal differences between songs or calls was typical of the differentiation observed between sympatric but related species. As a result, the benchmark of three diagnosable differences was considered a good “point of reference” for assigning species rank to allopatric populations in the same family. Diagnostically distinct populations not meeting this standard are ranked as subspecies under this model (Remsen 2010). Donegan and Avendaño (2008) applied this method to the tapaculos (Rhinocryptidae) and found examples of sympatric species that differed by only one, not three, diagnosably distinct vocal characters. This suggested that vocal benchmarks cannot be applied universally to all birds, even those in quite closely related families.

When species rank is assessed across a taxonomic group as a whole, consistency is a virtue. Under a biological species concept-based approach, attaining such consistency will require a determination of which allopatric populations have differentiated to the same extent as related sympatrics and which have not. Those that have so differentiated are species; those that have not are, at most, subspecies. Unfortunately, consistency is not attained in current classifications, especially as regards more diverse tropical faunas. This is generally due to discrepancies in available data, the regularity of different genera being revised and differences in approaches by regional committees or textbook authorities to studies using different taxonomic methods (e.g., molecular vs. morphological) (Sangster 2014, Donegan et al. 2015, Collar et al. 2016). Even in a popular group such as birds, in the tropics there are many more species and subspecies than there are taxonomists, meaning that only a small number of groups have been subject to modern studies. However, inconsistencies and stasis are compounded by biases of some taxonomic committees towards keeping “status quo” treatments of previous authorities, ahead of reflecting the results of modern reviews in certain publications (e.g., the field guide literature or less-prestigious journals) (Donegan et al. 2015). Large numbers of allopatric populations inhabiting different mountain ranges, lowland regions or islands lack modern studies to assess their rank, or studies may exist which have been ignored, and taxonomies as a whole are often based on tradition more than rationality.

Helbig et al. (2002)’s scheme for the comparative assessment of sympatric species has been applied by some taxonomic committees in Europe as the basis for splitting of a number of questionably valid species (e.g., Carrion Crow *Corvus corone* from Hooded Crow *Corvus cornix*: Parkin et al. 2003; American Herring Gull *Larus smithsonianus* from European Herring Gull *Larus argentatus*: Collinson et al. 2008). The former two crows are well-known to hybridize and establish relatively narrow contact zones where intermediate plumages prevail. Their split relies in part on a marginal bias towards non-crossing mate choice in such zones (Parkin et al. 2003). The latter two gulls have been considered diagnosable in immature plumages and mtDNA, but they have yet been found to be fully diagnosable in any adult plumage character and infrequent hybridization between allopatric related species obscures any interpretation of molecular results (Lonergan and Mullarney 2004, Sonsthagen et al. 2016), whilst voice has not yet been subject to detailed statistical analyses demonstrating diagnosability.

Neither of these two splits is problematic from a phylogenetic species concept or “enthusiastic splitter” perspective in isolation; and further studies could give stronger support to these treatments. However, based on my experience of working with birds in the Neotropics, the benchmark applied to these situations would result in the specific recognition of probably several thousands of current subspecies or unnamed taxa occurring in that region. Barrowclough et al. (2016) estimated that the number of recognized bird species globally would almost double, were phylogenetic species concepts to be applied. That factor would increase further under models that treat populations with non-diagnosable adults, such as the Herring Gulls referred to above, as species. Discrepancies arise because, at the same time as Europe’s leading taxonomic committees embarked on a program of enthusiastic splitting, countless diagnosable allopatric populations in the tropics that exhibit more considerable vocal or morphological differentiation (some of which have been shown by molecular studies not to be sister taxa or which barely resemble one another in voice or morphology) remain lumped by the more conservative taxonomic authorities addressing those regions. The current status of global bird taxonomies is, therefore, highly irrational and subject to regional bias.

Tobias et al. (2010) highlighted the internal inconsistency of avian taxonomies on a global scale and the lack of a universal framework for species delimitation. They proposed a universal “species scoring” test for assessing the taxonomic rank of birds. This takes into account not just vocal characters (as is broadly the case under the Isler et al. 1998 model) but also plumage, biometrics, sympatry/parapatry, hybridization, habitat, and ecology. Their system is based upon a series of scores of 0–4 for a maximum number of characters in particular categories. Differences are classified as minor (1) medium (2), major (3) or exceptional (4). For plumage, various guidelines were proposed for a judgement-based assessment. For continuous variables, Tobias et al. (2010) measured pooled effect sizes without controlling for sample sizes using *t*-distributions. In their system, populations showing 0.2–2 effect size difference (minor to below 50% diagnosability) score 1 point, 2–5 effect sizes (equivalent to 50% to >95+% diagnosability depending on sample size) score 2 points, those at 5–10 effect sizes score 3 and >10 score 4. This system was developed based on a study of 58 pairs of closely related sympatric species from 29 families. Del Hoyo and Collar (2014, 2016) applied the Tobias et al. (2010) system to all birds in a major book series, proposing over 400 splits and 20 lumps in the first edition alone.

The Tobias et al. (2010) method and outcomes of Del Hoyo and Collar (2014, 2016)’s new taxonomy have been criticized, on conceptual and organizational grounds (Remsen 2015, 2016, Bakker 2015, Garnett and Christidis 2017). Many of Del Hoyo and Collar (2014, 2016)’s South American splits were however supported by a critical review, although not in Toucans, a group that shows extraordinary intra-specific variation where species scoring produced unsupportable outcomes (Donegan et al. 2015). Although there have been calls for proposed new taxonomies in the work to be rejected (Remsen 2015) or restricted to situations where significant data gaps exist (Remsen 2016), some authors have reviewed the proposals and accepted or rejected them on a case-by-case basis (e.g., Donegan et al. 2015, Gill and Donsker 2018).

Garnett and Christidis (2017) criticized the “anarchy” in current taxonomy, citing the large number of splits by Del Hoyo and Collar (2014, 2016) and calling for the regulation by committee of splitting and lumping in taxonomy and moves to “restrict the freedom of taxonomic action”. This proposal has itself been widely criticized (e.g., Thomson et al. 2018, Collar 2018), some authors commenting that it “conflict[s] with some basic and indisputable principles underpinning the philosophy of science” (Raposo et al. 2017). There appears to be broad disagreement as to whether existing taxonomies are either (i) well-developed, only to be changed following review of the scientific literature by appropriately appointed persons; or (ii) irrational and in need of expeditious root-branch review. Those in both camps have claimed that the needs of conservation support their approach (Garnett and Christidis 2017, Collar et al. 2016). I have argued elsewhere that we are “fiddling while Rome burns, if being closed-minded to new findings that may challenge preconceptions or requiring perfect data sets for change”, in this era of extinctions (Donegan et al. 2015). Regardless of who has the best ideas about the politics of how taxonomy is organized, it can be said that *all* these modern controversies have a single underlying cause, namely the “allopatric problem” of species: how assessments are to be made, whether it matters that this is considered consistently, how urgent any reassessment is, what the right benchmark is and which persons or bodies are properly qualified to make the decisions.

In light of the difficulties with scoring “systems” and other developments, Halley et al. (2017) have argued for a return to monophyly and essentially Cracraft (1983)’s scheme as the basis for determining species rank for allopatrics. They cite the lack of a broadly supported universal benchmark test, the difficulty of finding sympatric sister groups for study and inconsistencies in existing taxonomies but also did not regard it as a problem that recognized allopatric versus sympatric species might show different levels of differentiation. Under such an approach, many named and unnamed subspecies occurring on different mountain ranges and islands in the tropics would be afforded species rank. Difficulties as to the appropriate setting of a benchmark in difficult cases are transferred from the “equivalent to a species” benchmark to a different point which distinguishes other borderline situations: i.e., claimed barely monophyletic versus claimed non-monophyletic groupings. Gill (2014) separately proposed that a null hypothesis of species rank should apply to some allopatric populations, but this proposal was criticized by Toews (2015). Such methods and approaches are not considered further here since, in the words of Halley et al. (2017), I am “philosophically tied to a yardstick approach”.

Over the last 20 years, I have been studying the taxonomy of birds in Colombia using biometric data (from mist-netting and museums) and using sound recordings. This resulted in the production of a large amount of data relevant to studying differentiation. It has become transparent to me that steps might be taken towards resolving some of these seemingly intractable fundamental disagreements, by developing an objective and agreeable basis, grounded in scientific method, statistics, the analysis of large data and based on traditional biological species concept thinking, that could be used better, more consistently and more rationally to assess the rank of allopatric populations. Ultimately, the aim of this study is to attempt definitively to provide a robust, objective and universal method to address the centuries-old question (unresolved since Poulton 1904), “What is a species?”, in the context of the allopatric problem and using real world data rather than molecular data.

## Materials and methods

In the present study, I took a large data set that had been developed for purposes of various particular taxonomic studies of birds (citations below) and used this to road-test proposed and possible alternative statistical tests for measuring differentiation or diagnosis, with the intention of studying outcomes of tests in order to inform recommendations.

I compiled vocal and biometric data from multiple studies, including of representatives of the three major assemblages of birds: non-passerines (three families), suboscine passerines (four families), and oscine passerines (two families) (citations in Tables 1–2). In all of these studies, an exhaustive approach was applied to obtaining relevant sound recordings from the world’s two largest avian sound recording repositories (as such databases stood prior to the point of publication): the xeno-canto.org collection and Macaulay Library, as well as commercially available CDs and DVDs and private sound recordings of the authors and other contacts. In relation to biometrics, most studies involved a relatively comprehensive set of available Colombian museum specimens, typically with over five and often more museums studied, including most of the main museums in Colombia, the USA, the UK, and France. For some studies, the largest Venezuelan collection was also studied. Full details of methods can be read in each relevant paper.

**Table 1.** Summary information on the vocal studies used in the analysis.

Order: Family	Genus	No. taxa / populations	No. spp. before review	No. spp. after review	No. continuous vocal variables	No. Pairwise tests omitted	Pairwise comparisons	Sample sizes (mean ± s.d.) (min–max)	Reference
Columbiformes: Columbidae	*Geotrygon*	2	1	2	2	0	2	22.0 ± 4.6 (18–26)	Donegan and Salaman (2012)
Apodiformes: Trochilidae	*Adelomyia*	2	1	1	10	0	10	15.7 ± 1.5 (14–18)	Donegan and Avendaño (2015)
Piciformes: Bucconidae	*Hypnelus*	2	1	2	5	0	5	5.5 ± 1.6 (4–7)	Donegan et al. (2015)
Passeriformes: Thamnophilidae	*Myrmeciza*	8	4	5	26	114	614	42.7 ± 49.2 (3–179)	Donegan (2012)
Passeriformes: Grallariidae	*Grallaricula*	10	1	2	14	224	406	18.2 ± 12.9 (3–63)	Donegan (2008)
Passeriformes: Rhinocryptidae	*Scytalopus 1*	8	3	3	12	0	336	23.0 ± 13.8 (4–57)	Donegan and Avendaño (2008)
Passeriformes: Rhinocryptidae	*Scytalopus 2*	2	1	1	7	0	7	14.9 ± 2.2 (12–17)	Donegan et al. (2013)
Passeriformes: Tyrannidae	*Sirystes*	4	1	4	18	64	44	39.1 ± 41.5 (3–146)	Donegan (2013)
Passeriformes: Parulidae	*Basileuterus*	13	3	6	19	558	924	25.5 ± 19.0 (2–78)	Donegan (2014)
	***TOTALS***	**51**	**16**	**26**	**113**	**960**	**2348**	**29.0** ± **32.5 (2**–**179)**	

**Table 2.** Summary information on the biometric studies used in the analysis.

Order: Family	Genus	No. taxa / populations	No. spp. before review	No. spp. after review	No. continuous biometric variables	No. Pairwise tests omitted	Pairwise comparisons	Sample sizes (mean ± s.d.) (min.–max.)	Reference
Apodiformes: Trochilidae	*Adelomyia*	2	1	1	4	0	4	9.6 ± 3.2(6–13)	Donegan and Avendaño (2015)
Passeriformes: Thamnophilidae	*Myrmeciza*	7	4	5	5	18	87	21.1 ± 19.5(2–65)	Donegan (2012)
Passeriformes: Grallariidae	*Grallaricula*	11	1	3	6	49	281	12.4 ± 9.3(3–37)	Donegan (2008)
Passeriformes: Rhinocryptidae	*Scytalopus 1*	8	4	3	5	31	109	8.7 ± 7.4(2–24)	Donegan and Avendaño (2008)
Passeriformes: Rhinocryptidae	*Scytalopus 2*	2	1	1	5	0	5	4.9 ± 2.3(3–9)	Donegan et al. (2013)
Passeriformes: Thraupidae	*Anisognathus*	10	2	2	5	39	186	25.1 ± 34.7(4–214)	Donegan and Avendaño (2010)
Passeriformes: Parulidae	*Basileuterus*	9	3	5	5	30	150	15.4 ± 10.9(2–42)	Donegan (2014)
	***TOTALS***	**49**	**16**	**20**	**35**	**167**	**822**	**15.5** ± **19.1(2**–**214)**	

Vocal variables always included measures of maximum acoustic frequency, length, number of notes and speed. In some studies, change in pace, minimum frequencies, frequencies of particular notes, note bandwidth, changes in acoustic frequency and position of peaks or troughs of frequency within a vocalization, or any of the same measures for particular parts of vocalizations, were also measured. In each study, the variables under study were designed so as to document as fully as possible observed subjective differences between populations. Biometric variables were in all cases wing, tail, tarsus and bill length and mass, except for Trochilidae (no tarsus length) and Grallariidae (where bill width was additionally measured). Note shape and other subjective vocal characters were also studied, as were plumages. However, information on non-continuous variables was discarded for purposes of this present study.

Pairwise comparisons were undertaken on a matrix basis of each population against each other population. Some pairwise tests were omitted due to lack of data for a particular population, i.e., where there were *n* < 2 recordings of a particular type of vocalization (which could represent either a sampling gap or genuine lack of delivery of such vocalization by the population in question); or *n* < 2 specimens of the population available in museums that were studied. In such cases, where *n* < 2, standard deviations could not be calculated and *t*-tests could not be run, so the comparison was excluded to ensure full comparability between all tests applied.

The data set was not designed for the study of statistical tests used in taxonomy, since this study had not been conceived at the time of data collection. The choice of taxonomic groups was not based only on studies which include among their components sympatric pairs (cf. Tobias et al. 2010). Necessarily, in those studies involving more than two populations, not all the populations undergoing pairwise comparisons are sisters of one another and, in some instances, subsequent molecular studies have demonstrated other (unstudied) taxa to be sister to some of the populations in the group under study. The distribution of study species is highly localized to north-western South America. The non-passerines part of the study set is much smaller than the passerines part. All studies involve situations where diversity appeared to have been previously underestimated at either species or subspecies level or both or followed a discovery of a new taxon whose taxonomic rank was investigated, resulting in a detailed study being undertaken. Most of the taxon pairs under comparison are subspecies/subspecies situations, and many of them involve populations that are not taxonomically recognized at all. For several populations, vocal studies were concluded without biometric data. For one study (*Anisognathus*), only biometric data were analyzed but not vocal data.

Several statistical tests were applied multiple times on a pairwise basis using a Microsoft Excel spreadsheet devised by the author for rapid assessment of multiple pairwise statistical tests across multiple populations. This spreadsheet is being published on the author’s researchgate.net page, and should assist authors in better and more swiftly analyzing diagnosability in future studies. Calculations, described below, were undertaken to measure inter-population differences in the context of various species and subspecies concepts.

First, the entire data set was subjected to various proposed tests of species or subspecies rank. In the formulae used below, *x̄*_1_ and s_1_ are the sample mean and standard deviations of Population 1; *x̄*_2_ and s_2_ refer to the same parameters in Population 2; and the t value uses a one-sided confidence interval at the percentage specified for the relevant population and variable, with t_1_ referring to Population 1 and t_2_ referring to Population 2.

LEVEL 1: Welch’s *t*-test at *p*<0.05/*n_v_*, i.e., applying a Bonferroni correction. An unequal variance (Welch’s) *t*-test was used. This is preferable to other *t*-tests in that it makes no assumptions about whether the SD of one population differs from that of the other. For vocal data potentially based on ratios, such as song speed, a two-sample Kolmogorov-Smirnov test can be applied instead to account for the possibility of a non-normal distribution. However, in order to standardize the study outputs, only Welch’s *t*-test was applied here.

When applying tests of statistical significance across multiple variables for the same pair, there is a risk of so-called “type 1” errors occurring. If testing for *p* < 0.05 for 100 independent variables of the same two populations, it would be expected that 5 variables would meet the requirements of the relevant test at this level of confidence. Various methods were tested which purport to reduce the risk of “type 1” errors. First, Bonferroni corrections were applied based on each of: (i) the total number of variables studied for the pair as a whole; (ii) separately for two “families” of vocal versus biometric variables; and (iii) separately for each different kind of vocalization, where applicable. Applying Bonferroni correction for a study involving five variables, *p* < (0.05/5) = 0.01 is the corrected confidence interval. Dunn-Šidák is a widely used but less conservative alternative to Bonferroni and was applied also to all three of the same situations as above in order to examine the impacts and outcomes using alternative corrections.

LEVEL 2: a ‘50%/95%’ test, following one of Hubbs and Perlmutter (1942)’s subspecies proposals but modified to control for sample size by using Isler et al. (1998)’s framework (see under Level 5) based on *t*-distribution. This test is passed if sample means are two average SDs or more apart controlling for sample size, i.e., the sample mean of each population falls outside the range of 95% of the other population:

|(*x̄*_1_–*x̄*_2_)| > (*s*_1_(*t*_1 @ 97.5%_) + *s*_2_(*t*_2 @ 97.5%_))/2

LEVEL 3: The traditional ‘75% / 99+%’ test for subspecies (Amadon 1949, Patten and Unitt 2002), modified to control for sample size, which requires both the following tests to be passed:

|(*x̄*_1_–*x̄*_2_)| > *s*_1_(*t*_1 @ 99%_) + *s*_2_(*t*_2 @ 75%_) and

|(*x̄*_2_–*x̄*_1_)| > *s*_2_(*t*_2 @ 99%_) + *s*_1_(*t*_1 @ 75%_)

LEVEL 4: diagnosability based on non-overlap of recorded values (the first part of Isler et al.’s 1998 diagnosability test).

LEVEL 5: ‘Full’ diagnosability (where sample means are four average SDs apart at the 95% level, controlling for sample size) the second part of Isler et al.’s (1998) diagnosability test:

|(*x̄*_1_–*x̄*_2_)| > *s*_1_(*t*_1 @ 97.5%_) + *s*_2_(*t*_2 @ 97.5%_)

**Figure 1.**
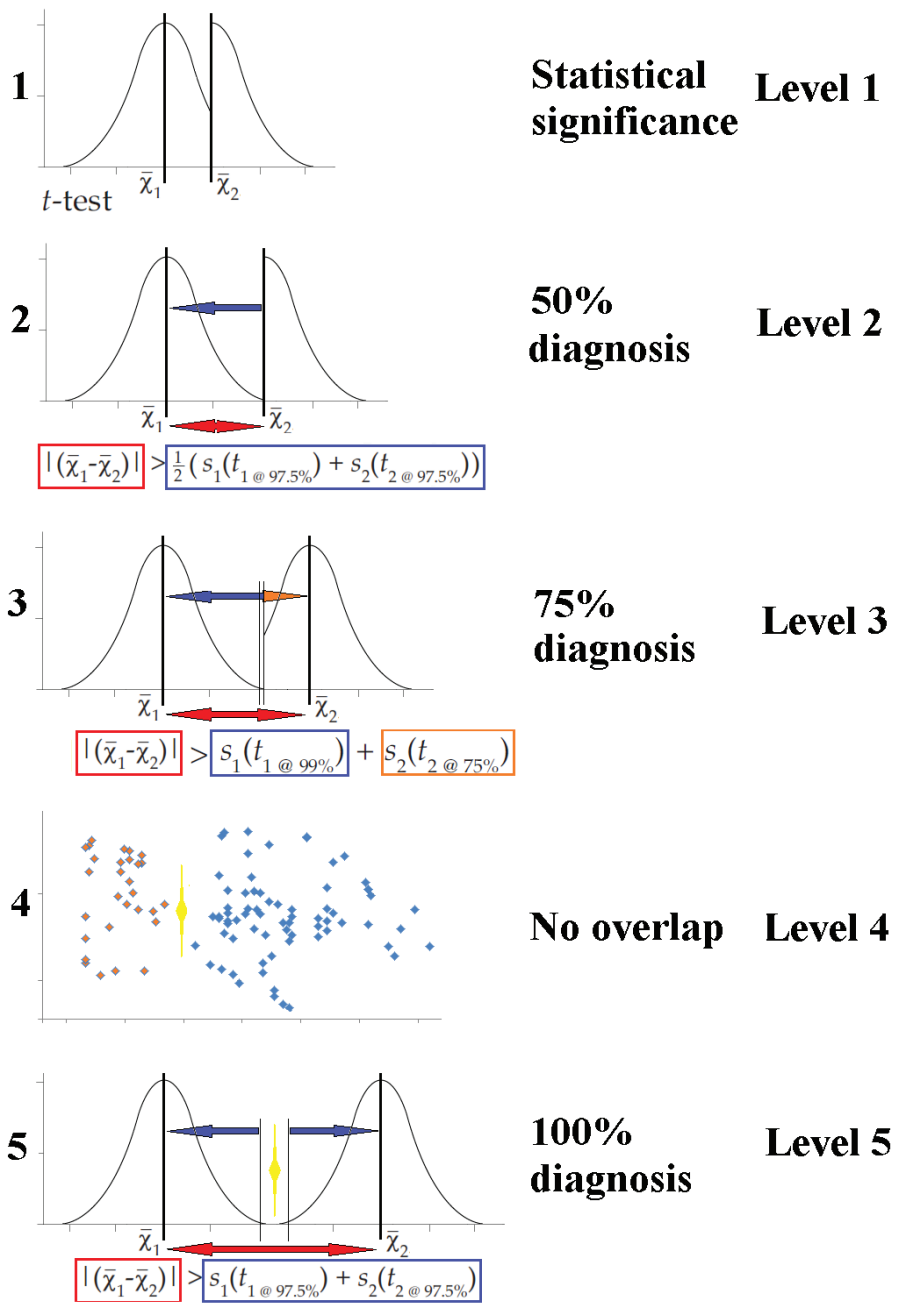
Graphical depiction of datasets which satisfy the Level 1–5 statistical tests addressed in this study.

Figure 1 illustrates how each of the Level 1 to Level 5 statistical tests measures differentiation.

These five tests were applied to 2348 population/variable combinations for voice and 822 population/variable combinations for biometrics. A population/variable combination is one comparison between two populations for a single variable. For example, in the *Grallaricula* study, a comparison of the main East Andes population against the Central Andes population for song length would constitute a single population/variable combination. With five diagnosability tests (Levels 1–5 above) conducted per population/variable combination, this means that a total of 15,610 pairwise statistical tests were run in this part of the study. (A further four tests conducted in later sections bring that total to over 28,000 separate statistical tests in this study.) Each population/variable combination was placed in a category summarizing which diagnosability tests it satisfied. The total number of population/variable combinations meeting particular tests was then summed for the biometric and vocal data sets separately, and then similar kinds of outcomes were grouped using the framework set out in Table 3. In order to consider taxonomic differences between the vocal and biometric data sets, data for studies involving the same taxonomic groups only are also presented.

**Table 3.** Recorded test satisfaction outcomes and the mapping of such outcomes to diagnosis groupings.

Outcome	Meaning	Grouping
0	None of the tests are met.	No diagnosis
1	Statistically significant difference between means but no tests of diagnosis are met and data overlap.	Statistical significance
14	Statistically significant difference between means and data show no overlap but no tests of diagnosis are met.	
12	Statistically significant difference between means but diagnosis only up to 50% and data overlap.	50% differentiation
124	Statistically significant difference between means, diagnosis up to 50% and data show no overlap.	
123	Statistically significant difference between means and diagnosis at both 50% and 75% levels but data overlap.	75% differentiation
1234	Statistically significant difference between means and diagnosis at both 50% and 75% levels and data do not overlap	
12345	Statistically significant difference between means and diagnosis at 50%, 75% and 95% levels and data do not overlap	95% differentiation
1235	Statistically significant difference between means and diagnosis at 50%, 75% and 95% levels but data overlap.	
1245	Statistically significant difference between means and diagnosis at 50% and 95% levels and data overlap but 75% test is not met.	
125	Statistically significant difference between means and diagnosis at 50% and 95% levels and data overlap but 75% test is not met and data overlap.	
2	No statistically significant difference between means, but 50% diagnosis test is met.	Possible false results
2345	No statistically significant difference between means, but 50%, 75% and 95% diagnosis tests are met.	
24	No statistically significant difference between means, but 50% diagnosis test is met and data do not overlap.	
245	No statistically significant difference between means, but 50% and 95% diagnosis tests are met and data do not overlap.	
25	No statistically significant difference between means and data overlap, but 50% and 95% diagnosis tests are met.	
4	Data do not overlap but no other statistical tests are met	

Certain minor methodological changes were undertaken here as compared to some of the underlying studies on which this paper is based: (i) where a single population had only one data point, it was excluded here from analyses, since only “Level 4” tests can be applied where degrees of freedom are 0 and this paper sought to compare outcomes for all comparisons; (ii) for the number of notes in the call for *Grallaricula*, several populations had uniformly one note in their calls, with standard deviation of zero, producing “divide by zero” errors for several tests, and so pairwise comparisons between such populations for that variable were excluded; (iii) some underlying studies presented biometric data for either males or females or all specimens or both; here, one or other of the “male” or “all specimens” data sets was selected, depending on whether material sexual differences in biometrics were observed and on sample size (generally, for studies with larger samples, using male only data is preferable, whilst in those studies with fewer specimens available, a combined data set was used here); (iv) for the main *Scytalopus* data set (Donegan and Avendaño 2008), whose study was accepted for publication during a formative stage of the development of the methods used here, the data sets needed amendment to apply some of the methods set out below; (v) Bonferroni correction for purposes of “Level 1” pass/fail analysis was applied based on number of vocal variables as a whole and not partitioned for different kinds of vocalization (this method ultimately being selected for reasons discussed later on); and (vi) only Welch’s *t*-tests (and no other “Level 1” tests used in the underlying studies) were applied, to promote comparability of outcomes. As a result, the results here differ in some instances from those found in the appendices to some of the papers it is based upon. Overall, these methodological changes result in differing numbers of positive outcomes at Levels 1 and 4 in particular, compared to those presented in the original publications. Also as a result of these changes, the entire data set was re-analyzed using Excel spreadsheets in order to produce comparisons and ensure reliable counting, with no reliance on previously published analyses of the same data.

### Effect sizes

The second part of this study aimed to measure effect sizes four different ways, in order to inform appropriate benchmarks for measuring or scoring differentiation. The impacts of using pooled standard deviations (as per Tobias et al. 2010), unpooled standard deviations (as per Isler et al. 1998) and of controlling for sample size using *t*-distribution (as per Isler et al. 1998) or not (as per Tobias et al. 2010) were compared.

### Bare unpooled effect sizes

Effect sizes were first calculated using the following formula:

|(*x̄*​_1_–​*x̄*​_2_)| /[(​*s*_1_+​​​*s*2)/2]​

This uses an arithmetic mean of the standard deviations of the two populations to measure the difference between the means of the same two populations.

### Controlled unpooled effect sizes

A control was applied using *t*-distribution values, following Isler et al. (1998), to produce a further set of effect size measurements:

|(*x̄*​​_1_–​​*x̄*​​_2_)| / ¼[*s*_1_(*t*_1 @ 97.5%_) + *s*_2_(*t*_2 @ 97.5%_)]

This measures the distance between the means of two populations in terms of numbers of SDs, but controlling for sample size using a *t* distribution. The factor of ¼ is included to maintain parity with bare unpooled effect sizes and other measures studied in this section, i.e., where mean differences are measured with the equivalent of a single standard deviation for their denominator. For a normal distribution, as *n* tends to infinity, *t* tends to *c.*2 (actually nearer to 1.98), capturing essentially the whole sample within 2 standard deviations. As a result, *s*_1_(*t*_1 @ 97.5%_) + *s*_2_(*t*_2 @ 97.5%_) is equivalent to 2*s*_1_ + 2*s*_2_, or 4*s*, calling for division by 4 to retain parity with 1*s*.

To illustrate the impact of this correction versus the results from using bare unpooled effect sizes, the maximum acoustic frequency in the “slow song” in Santa Marta Warbler *Basileuterus basilicus* differs from that in the East Andes population of Three-striped Warbler *B. tristriatus* by 4.087 SDs, using bare unpooled effect sizes. The controlled unpooled effect size for this variable is lower at 3.910 SDs. This is because *n* = 9 for *basicilus* and *n* = 53 for East Andes *tristriatus*; one-sided *t*-distribution values at 97.5% are 2.306 and 2.007 respectively, effectively reflecting that an average SD of 2.157 using these sample sizes is equivalent to an SD of *c.*2 with infinite data points). This particular population/variable comparison therefore moved from being in a diagnosable category (> 4 SDs’ difference) to *not* being diagnosable (< 4 SDs’ difference and failing Isler et al. 1998’s diagnosability test), when controlling for sample size.

### Bare pooled effect sizes

Effect sizes using a pooled standard deviation, or Cohen’s *d*, were calculated. First, the pooled standard deviation was calculated:

*s*_p_ = √[((*n*_1_–1)*s*_2_^2^ + (*n*_2_–1)*s*_2_^2^))/(*n*_1_+*n*_2_–2)]

Cohen’s *d* was then calculated as:

|(*x̄*_1_-*x̄*_2_)| / *s*_p_.

or, in full:

|(*x̄*_1_-​​*x̄*​​_2_)| / √[((*n*_1_–1)*s*_2_^2^ + (*n*_2_–1)*s*_2_^2^))/(*n*_1_+*n*_2_–2)]

This was the measure of effect size used by Tobias et al. (2010) and is used widely in science.

### Controlled pooled effect sizes

Bare pooled effect sizes were subjected to an equivalent control for sample size (as for bare unpooled effect sizes), but using *t*-values at the degrees of freedom of the pooled standard deviation:

Cohen’s *d* / ((*t*_pooled @ 97.5_)/2),

Where *t*_pooled_ is based on the degrees of freedom for the pooled standard deviation: d.f.=*n*_1_+*n*_2_–2.

or, in full:

|(*x̄*_1_–*x̄*_2_)| / (√[((*n*_1_–1)*s*_2_^2^ + (*n*_2_–1)*s*_2_^2^))/(*n*_1_+*n*_2_–2)]) / ((*t*_pooled@97.5%_)/2).

### Effect size buckets

Thee four measures of effect sizes were calculated for each population/variable combination and each outcome was then placed into two sets of buckets. First, in order to obtain a general resolution on effect sizes magnitude in taxonomic studies, population/variable combinations were placed into a set of buckets divided at 2 effect sizes (i.e., at approximately 50% differentiation) intervals: 0–2, 2–4, 4–6, etc. A second set of buckets was based on Tobias et al. (2010)’s scheme categories: character differences with an effect size of 0–0.2, 0.2–2; 2–5; 5–10 and >10.

### Plots and correlations

To compare the outcomes achieved using the four different measures of effect size and analyses of Levels 1–5, plots were produced between several of the outcomes. Spearman’s rank correlation coefficient was calculated as between statistical significance and effect size outcomes, based on the entire vocal and biometric data sets, so as to examine the inter-relation between the outcomes of applying different measures of differentiation.

## Results

### Type 1 correction analysis

Tables 4–5 illustrate the impacts on positive outcomes for statistical significance tests when applying different kinds of “type 1 correction”. The data also provide more detailed information on “Level 1” diagnosis (on which see further Tables 6–9 and “Levels analysis” below). For vocal variables (Table 4), over 70% of pairwise comparisons passed the Level 1 test using *p*<0.05. However, almost 15% of positive outcomes were eliminated when using the most conservative correction. The greatest impact among the cascade of tested corrections was to correct for sample size at all, which eliminated over 9% of positive outcomes. Fewer than 5% of outcomes were eliminated by treating all vocal variables as linked. The final impact on vocal data, treating all biometrics and voice as part of the same family of variables, affected <1.3% of outcomes. Generally speaking, Dunn-Šidák corrections had virtually nil impact compared to Bonferroni, with only five individual movements (<0.2%) from significant to non-significant categories across the entire set of vocal comparisons.

**Table 4.** Effect of applying different “Type 1 error” corrections on the vocal data set. The tests are ordered (A-G) from least to most conservative corrections. *Sirystes*, *Geotrygon* and *Hypnelus* data are presented outside the totals, since there were no biometric data set on which more conservative cumulative corrections could be applied. In the case of the latter two genera, *Adelomyia* and *Scytalopus 2*, only one kind of vocalization was studied.

	*Adelomyia*	*Myrmeciza*	*Grallaricula*	*Scytalopus 1*	*Scytalopus 2*	*Basileuterus*	TOTALS	[*Sirystes*]	[*Geotrygon*]	[*Hypnelus*]
**No. of vocal variables**	10	26	14	12	7	19		18	2	5
**A. No correction**										
*p*<	0.05	0.05	0.05	0.05	0.05	0.05		0.05	0.05	0.05
Passed	2	406	323	236	4	240	1211	32	2	2
Total	10	614	406	336	7	343	1716	44	2	5
% passed	*20*%	*66.1*%	*79.6*%	*70.2*%	*57.1*%	*70.0*%	***70.6***%	*72.7*%	*100*%	*40*%
**B. Dunn-Šidák with each kind of vocalisation separately**										
*p*<	0.00512	0.00639	0.00730	0.00851	0.00730	0.00730		0	0.0253	0.0102
Passed	2	357	293	197	3	200	1052	25	2	1
Total	10	614	406	336	7	343	1716	44	2	5
%	*20*%	*58.1*%	*72.2*%	*58.6*%	*42.9*%	*58.3*%	***61.3***%	*56.8*%	*100*%	*20*%
**C. Bonferroni with each kind of vocalisation separately**										
*p*<	0.005	0.00625	0.00714	0.00833	0.00714	0.00714286		0.01	0.025	0.01
Passed	2	357	293	197	3	200	1052	25	2	1
Total	10	614	406	336	7	343	1716	44	2	5
%	*20*%	*58.1*%	*72.2*%	*58.6*	*42.9*%	*58.3*%	***61.3***%	*56.8*%	*100*%	*20*%
**D. Dunn-Šidák with voice and biometrics separately**										
*p*<	0.00512	0.00197	0.00366	0.00427	0.00730	0.00730		0.00285	0.0253	0.0102
Passed	2	321	267	186	3	189	968	20	2	1
Total	10	614	406	336	7	343	1716	44	2	5
%	*20*%	*52.2*%	*65.8*%	*55.3*%	*42.9*%	*55.1*%	***56.4***%	*45.4*%	*100*%	*20*%
**E. Bonferroni with voice and biometrics separately**										
*p*<	0.005	0.00192	0.00357	0.00417	0.00714	0.00714		0.00278	0.025	0.01
Passed	2	321	266	185	3	188	965	20	2	1
Total	10	614	406	336	7	343	1716	44	2	5
%	*20*%	*52.2*%	*65.5*%	*55.1*%	*42.9*%	*54.8*%	***56.2***%	*45.5*%	*100*%	*20*%
**F. Dunn-Šidák: biometrics plus voice**										
*p*<	0.00366	0.00165	0.00256	0.00301	0.00427	0.00427				
Passed	2	317	260	179	3	182	943			
Total	10	614	406	336	7	343	1716			
%	*20*%	*51.6*%	*64.0*%	*53.3*%	*42.9*%	*53.1*%	***55.0***%			
**G. Bonferroni: biometrics plus voice**										
*p*<	0.00357	0.00161	0.0025	0.00294	0.00417	0.00417				
Passed	2	317	260	178	3	181	941			
Total	10	614	406	336	7	343	1716			
%	*20*%	*51.6*%	*64.0*%	*53.0*%	*42.9*%	*52.8*%	***54.8***%			

**Table 5.** Effect of applying different “Type 1 error” corrections on the biometric data set. The tests are ordered (A–E) from least to most conservative corrections. *Anisognathus* data are presented outside the totals, since there was no vocal data set on which more conservative cumulative corrections could be applied.

	*Adelomyia*	*Myrmeciza*	*Grallaricula*	*Scytalopus 1*	*Scytalopus 2*	*Basileuterus*	TOTALS	[*Anisognathus*]
**No. of biometric variables**	4	5	6	5	5	5		5
**A. No correction**								
*p*<	0.05	0.05	0.05	0.05	0.05	0.05		0.05
Passed	0	46	142	45	3	66	**302**	88
Total	4	87	281	109	5	150	**636**	186
% passed	*0*%	*52.9*%	*51.8*%	*41.3*%	*60*%	*44*%	***47.5***%	*47.3*%
**B. Dunn-Šidák with biometrics and voice separately**								
*p*<	0.0127	0.0102	0.00851	0.0102	0.0102	0.0102		0.0102
Passed	0	31	108	35	2	50	**226**	66
Total	4	87	281	109	5	150	**636**	186
%	*0*%	*35.6*%	*38.4*%	*32.1*%	*40*%	*33.3*%	***35.5***%	*35.5*%
**C. Bonferroni with biometrics and voice separately**								
*p*<	0.0125	0.01	0.00833	0.01	0.01	0.01		0.01
Passed	0	31	108	35	2	50	**226**	66
Total	4	87	281	109	5	150	**636**	186
%	*0*%	*35.6*%	*38.4*%	*32.1*%	*40*%	*33.3*%	***35.5***%	*35.5*%
**D. Dunn-Šidák: biometrics plus voice**								
*p*<	0.00366	0.00165	0.00256	0.00301	0.00427	0.00213		
Passed	0	33	92	31	2	40	**198**	
Total	4	87	281	109	5	150	**636**	
%	*0*%	*37.9*%	*32.7*%	*28.4*%	*40*%	*26.7*%	***31.1***%	
**E. Bonferroni: biometrics plus voice**								
*p*<	0.00357	0.00161	0.0025	0.00294	0.00417	0.00208		
Passed	0	33	92	31	2	40	**198**	
Total	4	87	281	109	5	150	**636**	
%	*0*%	*37.9*%	*32.7*%	*28.4*%	*40*%	*26.7*%	***31.1***%	

In the biometrics study, lower levels of statistically significant differentiation were found than for voice. More comparisons were non-significant (52.5%) than significant, even prior to applying any type 1 corrections. Applying type 1 corrections eliminated a further 12–16% of outcomes. Fewer than 5% of these eliminations result from treating voice and biometrics together; the bulk resulted from applying Bonferroni on the biometric data set itself. Dunn-Šidák corrections had no impact compared to using Bonferroni.

### Levels analysis

Tables 6–9 summarize the outcomes of diagnosis tests using the “Levels 1–5” model. After grouping the data, three main categories of positive diagnosis were revealed across the two studies, for both biometrics and voice: statistically significant, 50% differentiation and 95% differentiation. The category for 75% differentiation represented < 2.5% of outcomes in both studies.

**Table 6.** Outcomes of pairwise comparisons for vocal characters, placed into the different categories recovered by testing statistical tests of Levels 1 through 5. 1 = statistically significant, 2 = 50% diagnosis, 3=75% diagnosis, 4 = actual value diagnosis, 5 = 95% diagnosis (as detailed further in methods). See Table 3 for further information on meaning of codes used here.

Voice: levels passed	None	1	14	12	124	123	1234	12345	1235	1245	125	2	2345	24	245	4
*Geotrygon*		1			1											
*Adelomyia*	8	2														
*Hypnelus*	3				1											1
*Myrmeciza*	284	38	53	35	23	9	4	109	11	2	37	2		5	2	
*Grallaricula*	118	92	1	47	27	7	5	71	3	12	1	2	12			8
*Scytalopus 1*	148	82		37	17	5	7	36	1			1		2		
*Scytalopus 2*	4				3											
*Sirystes*	22	7		7	3		1	2				1			1	
*Basileuterus*	504	188	3	73	53	7	6	47	7	2	0	1		11	0	22
**TOTAL**	**1091**	**410**	**57**	**199**	**128**	**28**	**23**	**265**	**22**	**16**	**38**	**7**	**12**	**18**	**3**	**31**
**Percentages**	*46.5*%	*17.5*%	*2.4*%	*8.5*%	*5.5*%	*1.2*%	*1.0*%	*11.3*%	*0.9*%	*0.7*%	*1.6*%	*0.3*%	*0.5*%	*0.8*%	*0.1*%	*1.3*%

**Table 7.** Outcomes of pairwise comparisons for biometric characters, placed into the different categories recovered by testing statistical tests of Levels 1 through 5. 1 = statistically significant, 2 = 50% diagnosis, 3 = 75% diagnosis, 4 = actual value diagnosis, 5 = 95% diagnosis (as detailed further in methods). See Table 3 for further information on meaning of codes used here.

Biometrics: levels passed	None	1	14	12	124	123	1234	12345	1235	1245	125	2	2345	24	245	4
*Adelomyia*	4															
*Myrmeciza*	25	24		9	1			4								24
*Grallaricula*	166	17	1	15	23	1	5	35		3				7		8
*Scytalopus 1*	47	6	4	8	12		2	3								27
*Scytalopus 2*	3				1			1								
*Anisognathus*	120	43		12	6		1	4								
*Basileuterus*	97	27	1	10	9		1	2						1		2
**TOTAL**	**462**	**117**	**6**	**54**	**52**	**1**	**9**	**49**	**0**	**3**	**0**	**0**	**0**	**8**	**0**	**61**
**Percentages**	*56.2*%	*14.2*%	*0.7*%	*6.6*%	*6.3*%	*0.1*%	*1.1*%	*6.0*%	*0.0*%	*0.4*%	*0.0*%	*0.0*%	*0.0*%	*1.0*%	*0.0*%	*7.4*%

**Table 8.** Outcomes of pairwise comparisons using Levels analysis, for voice, by grouping. See Table 3 for information on Levels groupings used for column labels. % comparable data includes only those data sets in which both biometrics and voice were studied.

Voice: Taxon	Pairwise statistical tests (/5)	No diff.	Poss. false results	Signif. Only	50%	75%	95%
*Geotrygon*	2	0	0	1	1	0	0
*Adelomyia*	10	8	0	2	0	0	0
*Hypnelus*	5	3	1	0	1	0	0
*Myrmeciza*	614	284	9	91	58	13	159
*Grallaricula*	406	118	22	93	74	12	87
*Scytalopus 1*	336	148	3	82	54	12	37
*Scytalopus 2*	7	4	0	0	3	0	0
*Sirystes*	44	22	2	7	10	1	2
*Basileuterus*	924	504	34	191	126	13	56
***TOTALS***	**2348**	**1091**	**71**	**467**	**327**	**51**	**341**
OVERALL %		*46.5*%	*3.0*%	*19.9*%	*13.9*%	*2.2*%	*14.5*%
% (comparable)		*46.4*%	*2.9*%	*20.0*%	*13.7*%	*2.1*%	*14.7*%

**Table 9.** Outcomes of pairwise comparisons using Levels analysis, for biometrics, by grouping. See Table 3 for information on Levels groupings used for column labels. % comparable data includes only those data sets in which both biometrics and voice were studied.

Biometrics: Taxon	Pairwise statistical tests (/5)	No diff.	Poss. false results	Signif. only	50%	75%	95%
*Adelomyia*	4	4	0	0	0	0	0
*Myrmeciza*	87	25	24	24	10	0	4
*Grallaricula*	281	166	15	18	38	6	38
*Scytalopus 1*	109	47	27	10	20	2	3
*Scytalopus 2*	5	3	0	0	1	0	1
*Anisognathus*	186	120	0	43	18	1	4
*Basileuterus*	150	97	3	28	19	1	2
**TOTALS**	**822**	**462**	**69**	**123**	**106**	**10**	**52**
OVERALL %		*56.2*%	*8.4*%	*15.0*%	*12.9*%	*1.2*%	*6.3*%
% (comparable)		*53.8*%	*10.8*%	*12.6*%	*13.8*%	*1.4*%	*7.5*%

As foreshadowed in the type 1 error analysis (Tables 4–5), “no diagnosis” was the largest segment in the voice study, albeit a minority overall. For biometrics, “no diagnosis” exceeded all other outcomes combined. Possible false results were < 3% for the vocal sample but rose to 8.4% for the biometric data set, mostly relating to instances of non-overlap (Level 4). Such outcomes are more frequent when dealing with the smaller sample sizes that are more regularly presented by studies of specimens (see Tables 1–2). Experience from the process of collecting data during the course of these studies and re-running analyses is that Level 4 differences in initial analyses will ultimately often convert into Level 1, 2, 3 or even 5 differences with a greater sample, whilst others will erode to nothing and will simply have reflected a clustering of data points.

Isler et al. (1998)’s gold standard of diagnosis was met by 14.5% of vocal pairwise comparisons and 6.3% of biometrics comparisons. Approximately triple this number of outcomes, a total of 36% (voice) and 29% (biometrics) of outcomes, involved non-diagnosable but statistically significant differentiation.

Levels 1–5 were generally ordered by least to most exacting in terms of difficulty to pass. However, several examples of “outliers” were uncovered, where more liberal test outcomes were apparently “skipped”, e.g.: (i) only statistical significance and non-overlap (1&4); (ii) statistical significance with 50% and non-overlap but not 75% diagnosis (124); (iii) all tests being passed except non-overlap (123&5); (iv) all tests including 95% diagnosis being passed, but excluding 75% diagnosis (124&5); (v) full statistical diagnosis and 50% and 95% diagnosis being met but neither 75% nor non-overlap (12&5); and (vi) combinations skipping statistical significance altogether, but passing other tests (all outcomes starting with 2 or 4). These outcomes are all statistically plausible, including as a result of the values of *t* at particular sample sizes for different confidence limits, even if in some cases they are logically counterintuitive.

In terms of specific findings for birds, biometric data were less informative than vocal data with “possibly false results” also being more frequent for biometric comparisons. Tobias et al. (2010) also found that vocal characters exhibit greater measured differentiation than biometric variables. The biometric data set rarely attained higher levels of diagnosability, with 75% and 95% outcomes around half those for vocal data. This pattern remains after controlling for taxonomy.

### Effect sizes by 2d

Results for effect sizes divided into buckets of 2*d* are set out in Tables 10–11 for each of the four effect size measures used in the study, in each case for both voice and biometrics. In all data sets, a predominance of low differentiation (0–2 effect sizes, or less than 50% differentiation) is evident. A considerable 63–74% of outcomes fell into this lowest category, with a gradual tailing off of outcomes at increasing levels of differentiation.

**Table 10.** Results of the effects size study for voice, partitioning the data into 2 effect size intervals. The top two tables are based upon actual standard deviations for each set of data subjected to pairwise comparison. The lower two tables are based on pooled standard deviation data pooling. In each case, “bare” effect sizes are shown first (above). The second and fourth tables use “controlled effect sizes” for the relevant pooling approach, calculated by taking into account *t*-distribution values for the relevant sample size (or pooled sample size).

Bare Unpooled Effect Sizes (Voice)											
**Taxon**	**0–2**	**2–4**	**4–6**	**6–8**	**8–10**	**10–12**	**12–14**	**14–16**	**16–18**	**18–20**	**20**+
*Geotrygon*	1	1									
*Adelomyia*	10										
*Hypnelus*	3	2									
*Myrmeciza*	361	119	55	33	11	14	8	6	6	1	0
*Grallaricula*	208	91	47	16	10	7	3	14	6	3	1
*Scytalopus 1*	227	66	30	7	6						
*Scytalopus 2*	4	3									
*Sirystes*	27	14	2	0	1						
*Basileuterus*	654	168	47	29	20	4	1	1	0	0	0
***TOTAL***	**1497**	**462**	**181**	**85**	**48**	**25**	**12**	**21**	**12**	**4**	**1**
*Percentage*	*63.7*%	*19.8*%	*7.7*%	*3.6*%	*2.0*%	*1.1*%	*0.5*%	*0.9*%	*0.5*%	*0.2*%	*0.0*%
**Controlled Unpooled Effect Sizes (Voice)**											
**Taxon**	**0–2**	**2–4**	**4–6**	**6–8**	**8–10**	**10–12**	**12–14**	**14–16**	**16–18**	**18–20**	**20**+
*Geotrygon*	1	1									
*Adelomyia*	10										
*Hypnelus*	4	1									
*Myrmeciza*	375	114	53	32	14	15	0	7	3	1	
*Grallaricula*	219	88	43	25	14	5	6	4	1	0	1
*Scytalopus 1*	230	69	27	7	3						
*Scytalopus 2*	4	3									
*Sirystes*	29	12	3								
*Basileuterus*	717	151	34	14	6	2					
***TOTAL***	**1589**	**439**	**160**	**78**	**37**	**22**	**6**	**11**	**4**	**1**	**1**
*Percentage*	*67.7*%	*18.7*%	*6.8*%	*3.3*%	*1.6*%	*0.9*%	*0.3*%	*0.5*%	*0.2*%	*0.0*%	*0.0*%
**Bare Pooled Effect Sizes (Voice)**											
**Taxon**	**0–2**	**2–4**	**4–6**	**6–8**	**8–10**	**10–12**	**12–14**	**14–16**	**16–18**	**18–20**	**20**+
*Geotrygon*	1	1									
*Adelomyia*	10										
*Hypnelus*	3	2									
*Myrmeciza*	371	130	48	19	17	10	9	4	6	0	0
*Grallaricula*	222	98	33	16	6	9	7	6	3	2	4
*Scytalopus 1*	232	58	36	5	5						
*Scytalopus 2*	4	3									
*Sirystes*	28	13	2	0	0	0	0	1			
*Basileuterus*	687	151	40	16	11	8	5	2	1	1	2
***TOTAL***	**1558**	**456**	**159**	**56**	**39**	**27**	**21**	**13**	**10**	**3**	**6**
*Percentage*	*66.4*%	*19.4*%	*6.8*%	*2.4*%	*1.7*%	*1.1*%	*0.9*%	*0.6*%	*0.4*%	*0.1*%	*0.3*%
**Controlled Pooled Effect Sizes (Voice)**											
**Taxon**	**0–2**	**2–4**	**4–6**	**6–8**	**8–10**	**10–12**	**12–14**	**14–16**	**16–18**	**18–20**	**20**+
*Geotrygon*	1	1									
*Adelomyia*	10										
*Hypnelus*	3	2									
*Myrmeciza*	374	127	49	21	19	6	8	4	6		
*Grallaricula*	226	99	31	17	8	8	6	5	2	2	2
*Scytalopus 1*	233	58	35	5	5						
*Scytalopus 2*	4	3									
*Sirystes*	28	13	2	0	0	0	1				
*Basileuterus*	694	153	31	22	8	7	4	1	1	1	2
***TOTAL***	**1573**	**456**	**148**	**65**	**40**	**21**	**19**	**10**	**9**	**3**	**4**
*Percentage*	*67.0*%	*19.4*%	*6.3*%	*2.8*%	*1.7*%	*0.9*%	*0.8*%	*0.4*%	*0.4*%	*0.1*%	*0.2*%

**Table 11.** Results of the effects size study for biometrics, partitioning the data into 2 effect size intervals. The top two tables are based upon actual standard deviations for each set of data subjected to pairwise comparison. The lower two tables are based on pooled standard deviation data. In each case, “bare” effect sizes are shown first (above). The second and fourth tables use “controlled effect sizes” for the relevant pooling approach, calculated by taking into account *t*-distribution values for the relevant sample size (or pooled sample size).

**Bare Unpooled Effect Sizes (Biometrics)**											
**Taxon**	**0–2**	**2–4**	**4–6**	**6–8**	**8–10**	**10–12**	**12–14**	**14–16**	**16–18**	**18–20**	**20**+
*Adelomyia*	4										
*Myrmeciza*	58	25	4								
*Grallaricula*	170	63	21	14	5	3	1	2	0	2	
*Scytalopus 1*	67	33	7	1	1						
*Scytalopus 2*	2	2	0	1							
*Anisognathus*	154	26	5	0	1						
*Basileuterus*	116	27	7								
***TOTAL***	**571**	**176**	**44**	**16**	**7**	**3**	**1**	**2**	**0**	**2**	**0**
*Percentage*	*69.5*%	*21.4*%	*5.4*%	*1.9*%	*0.9*%	*0.4*%	*0.1*%	*0.2*%	*0.0*%	*0.2*%	*0.0*%
**Controlled Unpooled Effect Sizes (Biometrics)**											
**Taxon**	**0–2**	**2–4**	**4–6**	**6–8**	**8–10**	**10–12**	**12–14**	**14–16**	**16–18**	**18–20**	**20**+
*Adelomyia*	4										
*Myrmeciza*	73	10	4								
*Grallaricula*	192	51	20	12	3	0	0	1	2		
*Scytalopus 1*	84	22	2	1							
*Scytalopus 2*	3	1	1								
*Anisognathus*	163	19	3	1							
*Basileuterus*	127	21	2								
***TOTAL***	**646**	**124**	**32**	**14**	**3**	**0**	**0**	**1**	**2**	**0**	**0**
*Percentage*	*78.6*%	*15.1*%	*3.9*%	*1.7*%	*0.4*%	*0.0*%	*0.0*%	*0.1*%	*0.2*%	*0.0*%	*0.0*%
**Bare Pooled Effect Sizes (Biometrics)**											
**Taxon**	**0–2**	**2–4**	**4–6**	**6–8**	**8–10**	**10–12**	**12–14**	**14–16**	**16–18**	**18–20**	**20**+
*Adelomyia*	4										
*Myrmeciza*	57	28	2								
*Grallaricula*	177	54	23	14	3	5	2	2	1		
*Scytalopus 1*	67	36	6								
*Scytalopus 2*	2	2	1								
*Anisognathus*	158	23	4	0	1						
*Basileuterus*	127	17	6								
***TOTAL***	**592**	**160**	**42**	**14**	**4**	**5**	**2**	**2**	**1**	**0**	**0**
*Percentage*	*72.0*%	*19.5*%	*5.1*%	*1.7*%	*0.5*%	*0.6*%	*0.2*%	*0.2*%	*0.1*%	*0.0*%	*0.0*%
**Controlled Pooled Effect Sizes (Biometrics)**											
**Taxon**	**0–2**	**2–4**	**4–6**	**6–8**	**8–10**	**10–12**	**12–14**	**14–16**	**16–18**	**18–20**	**20**+
*Adelomyia*	4										
*Myrmeciza*	58	27	2								
*Grallaricula*	181	54	21	11	8	3	1	1	1		
*Scytalopus 1*	75	30	4								
*Scytalopus 2*	3	2									
*Anisognathus*	161	21	3	0	1						
*Basileuterus*	128	17	5								
***TOTAL***	**610**	**151**	**35**	**11**	**9**	**3**	**1**	**1**	**1**	**0**	**0**
*Percentage*	*74.2*%	*18.4*%	*4.3*%	*1.3*%	*1.1*%	*0.4*%	*0.1*%	*0.1*%	*0.1*%	*0.0*%	*0.0*%

A good portion (15–22%) of outcomes fell into the 2–4 effect sizes category, which, when using controlled unpooled effect sizes, corresponds to Level 2 in Tables 5–6. Outcomes in this bucket exceeded the total number of outcomes across all higher diagnosability categories. Even after applying the most conservative effect size calculations, very large effect sizes of over 20 were recorded in a handful of instances. Outcomes in all categories above 4–6 (inclusive) using controlled unpooled effect sizes correspond to the number of outcomes meeting Isler et al. (1998)’s diagnosis test (Level 5 in Tables 6–7), which is based on a score of 4*d* or more.

Table 12 illustrates the impact of applying increasingly more conservative tests of effect size using the 2*d* analysis, which is discussed further under “Pooled versus unpooled and bare versus controlled effect sizes” below.

**Table 12.** Changes in effect size categories resulting from increasingly more conservative tests of effect size being applied. This table is based upon changes between the categories in Tables 10–11.

**Voice: changes into effect size category**	**0–2**	**2–4**	**4–6**	**6–8**	**8–10**	**10–12**	**12–14**	**14–16**	**16–18**	**18–20**	**20**+
Bare Unpooled –> Bare Pooled	+63	-8	-22	-29	-9	+2	+9	-8	-2	-1	+5
Bare Pooled –> Controlled Pooled	+15	0	-11	+9	+1	-6	-2	-3	-1	0	-2
Controlled Pooled –> Controlled Unpooled	+16	-17	+12	+13	-3	+1	-13	+1	-5	-2	-3
**Total change from Bare Unpooled –> Controlled Unpooled**	+**94**	-**25**	-**21**	-**7**	-**11**	-**3**	-**6**	-**10**	-**8**	-**3**	**0**
As percentage of total	+*4.0*%	-*1.1*%	-*0.9*%	-*0.3*%	-*0.5*%	-*0.1*%	-*0.3*%	-*0.4*%	-*0.3*%	-*0.1*%	*0.0*%
**Biometrics: changes into effect size category**	**0–2**	**2–4**	**4–6**	**6–8**	**8–10**	**10–12**	**12–14**	**14–16**	**16–18**	**18–20**	**20**+
Bare Unpooled –> Bare Pooled	+21	-16	-2	-2	-3	+2	+1	0	+1	-2	0
Bare Pooled –> Controlled Pooled	+18	-9	-7	-3	+5	-2	-1	-1	0	0	0
Controlled Pooled –> Controlled Unpooled	+36	-27	-3	+3	-6	-3	-1	0	+1	0	0
**Total change from Bare Unpooled –> Controlled Unpooled**	+**75**	-**52**	-**12**	-**2**	-**4**	-**3**	-**1**	-**1**	+**2**	-**2**	**0**
As percentage of total	+*9.1*%	-*6.3*%	-*1.5*%	-*0.2*%	-*0.5*%	-*0.4*%	-*0.1*%	-*0.1*%	+*0.2*%	-*0.2*%	*0.0*%

### Effect sizes by Tobias et al. (2010) categories

Results for effect sizes divided into Tobias et al. (2010) bucket categories are set out in Tables 13–14 for each of the four effect size measures used in the study, in each case for both voice and biometrics. In contrast to the Levels 1–5 analysis, where “no diagnosis” predominated, or the 2*d* buckets, where the lowest category was largest, an overwhelming proportion (87–91%) of outcomes scored 1 or more under this system. The 3-point threshold of 5 or more effect sizes returned fewer positive scores (3.8–11.9%) than the Level 5 (or Isler et al. 1998) test of diagnosability or the total of elements in 2*d* buckets over 4 effect sizes. Overall, 77–85% of outcomes scored 1 or 2 points.

**Table 13.** Results of the effects size study for voice using Tobias et al. (2010) categories. The top two tables are based upon actual standard deviations for each set of data subjected to pairwise comparison. The lower two tables are based on pooled standard deviation data. In each case, “bare” effect sizes are shown first (above). The second and fourth tables use “controlled effect sizes” for the relevant pooling approach, calculated by taking into account *t*–distribution values for the relevant sample size (or pooled sample size).

**Bare Unpooled Effect Sizes (Voice)**					
**Taxon**	**0–0.2**	**0.2–2**	**2–5**	**5–10**	**10**+
*Geotrygon*	0	1	1		
*Adelomyia*	3	7			
*Hypnelus*	0	3	2		
*Myrmeciza*	70	291	152	66	35
*Grallaricula*	34	174	119	45	34
*Scytalopus 1*	31	196	83	26	
*Scytalopus 2*	0	4	3		
*Sirystes*	4	23	14	3	
*Basileuterus*	96	558	200	64	6
***TOTAL***	**239**	**1257**	**574**	**204**	**75**
*Percentage*	*10.1*%	*53.5*%	*24.4*%	*8.7*%	*3.2*%
**Controlled Unpooled Effect Sizes (Voice)**					
**Taxon**	**0–0.2**	**0.2–2**	**2–5**	**5–10**	**10**+
*Geotrygon*	0	1	1		
*Adelomyia*	3	7			
*Hypnelus*	0	4	1		
*Myrmeciza*	73	302	144	69	26
*Grallaricula*	37	182	121	49	17
*Scytalopus 1*	33	197	85	21	
*Scytalopus 2*	0	4	3		
*Sirystes*	4	25	12	3	
*Basileuterus*	113	604	171	34	2
***TOTAL***	**263**	**1326**	**538**	**176**	**45**
*Percentage*	*11.2*%	*56.5*%	*22.9*%	*7.5*%	*1.9*%
**Bare Pooled Effect Sizes (Voice)**					
**Taxon**	**0–0.2**	**0.2–2**	**2–5**	**5–10**	**10**+
*Geotrygon*	0	1	1		
*Adelomyia*	3	7			
*Hypnelus*	0	3	2		
*Myrmeciza*	71	300	157	57	29
*Grallaricula*	35	187	118	35	31
*Scytalopus 1*	31	201	78	26	
*Scytalopus 2*	0	4	3		
*Sirystes*	4	24	14	2	
*Basileuterus*	105	582	181	37	19
***TOTAL***	**249**	**1309**	**554**	**157**	**79**
*Percentage*	*10.6*%	*55.7*%	*23.6*%	*6.7*%	*3.4*%
**Controlled Pooled Effect Sizes (Voice)**					
**Taxon**	**0–0.2**	**0.2–2**	**2–5**	**5–10**	**10**+
*Geotrygon*	0	1	1		
*Adelomyia*	3	7			
*Hypnelus*	0	3	2		
*Myrmeciza*	71	303	158	58	24
*Grallaricula*	36	190	115	40	25
*Scytalopus 1*	30	203	79	24	
*Scytalopus 2*	0	4	3		
*Sirystes*	4	24	14	2	
*Basileuterus*	109	585	177	37	16
***TOTAL***	**253**	**1320**	**549**	**161**	**65**
*Percentage*	*10.8*%	*56.2*%	*23.4*%	*6.9*%	*2.8*%

**Table 14.** Results of the effects size study for biometrics using Tobias et al. (2010) categories. The top two tables are based upon actual standard deviations for each set of data subjected to pairwise comparison. The lower two tables are based on pooled standard deviation data. In each case, “bare” effect sizes are shown first (above). The second and fourth tables use “controlled effect sizes” for the relevant pooling approach, calculated by taking into account *t*-distribution values for the relevant sample size (or pooled sample size).

**Bare Unpooled Effect Sizes (Biometrics)**					
**Taxon**	**0–0.2**	**0.2–2**	**2–5**	**5–10**	**10**+
*Adelomyia*	1	3			
*Myrmeciza*	5	53	27	2	
*Grallaricula*	24	146	70	33	8
*Scytalopus 1*	7	60	40	2	
*Scytalopus 2*	0	2	2	1	
*Anisognathus*	22	132	28	4	
*Basileuterus*	19	97	32	2	
***TOTAL***	**78**	**493**	**199**	**44**	**8**
*Percentage*	*9.5*%	*60.0*%	*24.2*%	*5.4*%	*1.0*%
**Controlled Unpooled Effect Sizes (Biometrics)**					
**Taxon**	**0–0.2**	**0.2–2**	**2–5**	**5–10**	**10**+
*Adelomyia*	1	3			
*Myrmeciza*	8	65	14		
*Grallaricula*	29	163	61	25	3
*Scytalopus 1*	17	67	24	1	
*Scytalopus 2*	0	3	2		
*Anisognathus*	24	139	21	2	
*Basileuterus*	28	99	23		
***TOTAL***	**107**	**539**	**145**	**28**	**3**
*Percentage*	*13.0*%	*65.6*%	*17.6*%	*3.4*%	*0.4*%
**Bare Pooled Effect Sizes (Biometrics)**					
**Taxon**	**0–0.2**	**0.2–2**	**2–5**	**5–10**	**10**+
*Adelomyia*	1	3			
*Myrmeciza*	6	51	28	2	
*Grallaricula*	26	151	63	31	10
*Scytalopus 1*	8	59	40	2	
*Scytalopus 2*	0	2	3		
*Anisognathus*	23	135	24	4	
*Basileuterus*	20	97	21	12	
***TOTAL***	**84**	**498**	**179**	**51**	**10**
*Percentage*	*10.2*%	*60.6*%	*21.8*%	*6.2*%	*1.2*%
**Controlled Pooled Effect Sizes (Biometrics)**					
**Taxon**	**0–0.2**	**0.2–2**	**2–5**	**5–10**	**10**+
*Adelomyia*	1	3			
*Myrmeciza*	7	51	28	1	
*Grallaricula*	30	151	63	31	6
*Scytalopus 1*	11	64	32	2	
*Scytalopus 2*	0	2	3		
*Anisognathus*	23	138	22	3	
*Basileuterus*	21	107	22		
***TOTAL***	**93**	**516**	**170**	**37**	**6**
*Percentage*	*11.3*%	*62.8*%	*20.7*%	*4.5*%	*0.7*%

Changes between category (Table 15) were reduced here compared to the 2*d* effect size analysis, reflecting the smaller number of diagnosability categories studied and their greater effect size ranges.

**Table 15.** Changes in Tobias et al. (2010) category resulting from increasingly more conservative tests of effect size being applied. This table is based upon changes between the categories in Tables 13–14.

**Voice: changes into effect size category**	**0–0.2**	**0.2–2**	**2–5**	**5–10**	**10**+
Bare Unpooled –> Bare Pooled	+11	+52	-20	-47	+4
Bare Pooled –> Controlled Pooled	+4	+11	-5	+4	-14
Controlled Pooled –> Controlled Unpooled	+10	+6	-11	+15	-20
**Total change fromBare Unpooled –> Controlled Unpooled**	+**25**	+**69**	-**36**	-**28**	-**30**
As percentage of total	*1.1*%	*2.9*%	-*1.5*%	-*1.2*%	-*1.3*%
**Biometrics: changes into effect size category**	**0–0.2**	**0.2–2**	**2–5**	**5–10**	**10**+
Bare Unpooled –> Bare Pooled	+6	+5	-20	+7	+2
Bare Pooled –> Controlled Pooled	+9	+18	-9	-14	-4
Controlled Pooled –> Controlled Unpooled	+14	+23	-25	-9	-3
**Total change fromBare Unpooled –> Controlled Unpooled**	+**29**	+**46**	-**54**	-**16**	-**5**
As percentage of total	*3.5*%	*5.6*%	-*6.6*%	-*1.9*%	-*0.6*%

### Pooled versus unpooled and bare versus controlled effect sizes

**Figure 2.**
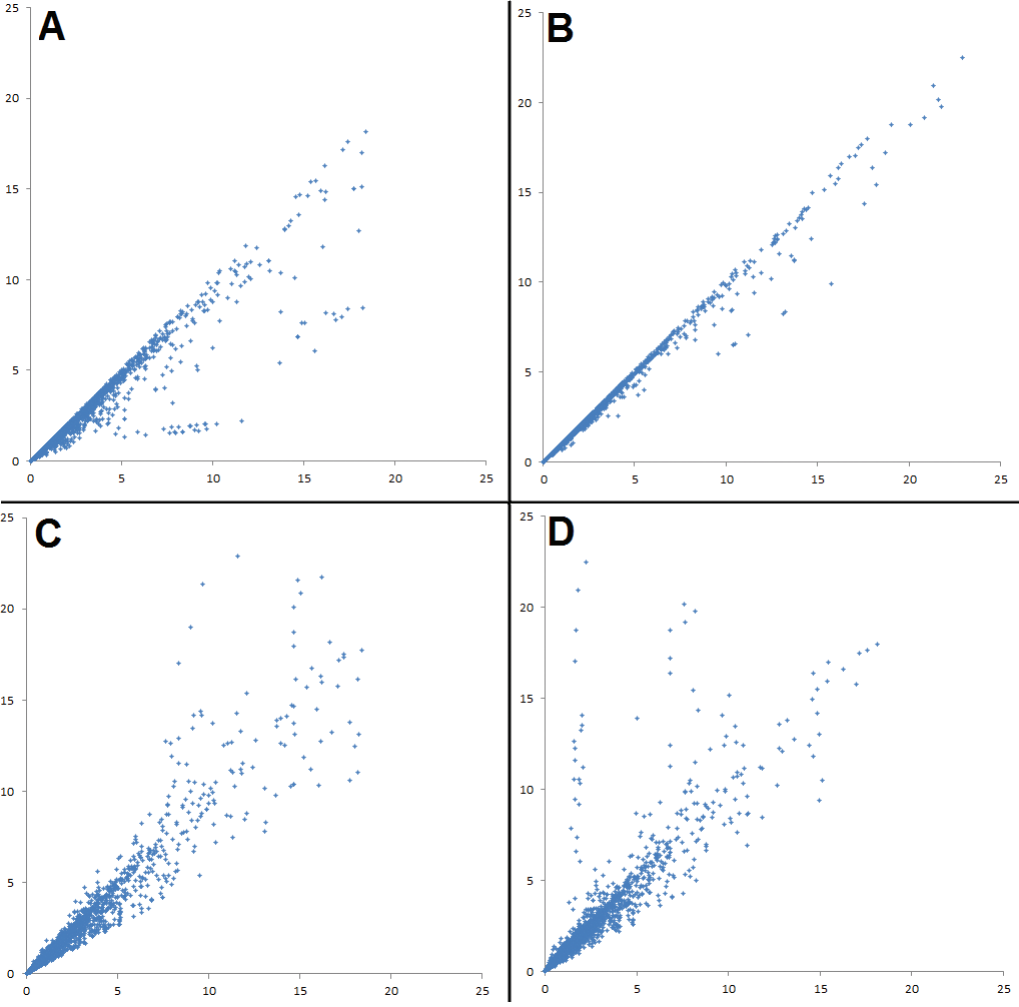
Scatter-graphs showing the effects of applying different corrections of effect size on the entire vocal data set. Each axis shows effect size, measured in a different way. **A** Controlling for sample size using unpooled data – *x*-axis: bare unpooled effect size; *y*-axis: controlled unpooled effect size **B** Controlling for sample size using pooled data – *x*-axis: bare pooled effect size; *y*-axis: controlled pooled effect size **C** Using pooled versus unpooled effect sizes without controlling for sample size – *x*-axis: bare unpooled effect size; *y*-axis: bare pooled effect size **D** Using pooled versus unpooled effect sizes and controlling for sample size – *x*-axis: controlled unpooled effect size; *y*-axis: controlled pooled effect size. A single data point of greater than 25 effect sizes was excluded to improve presentation of the results.

**Figure 3.**
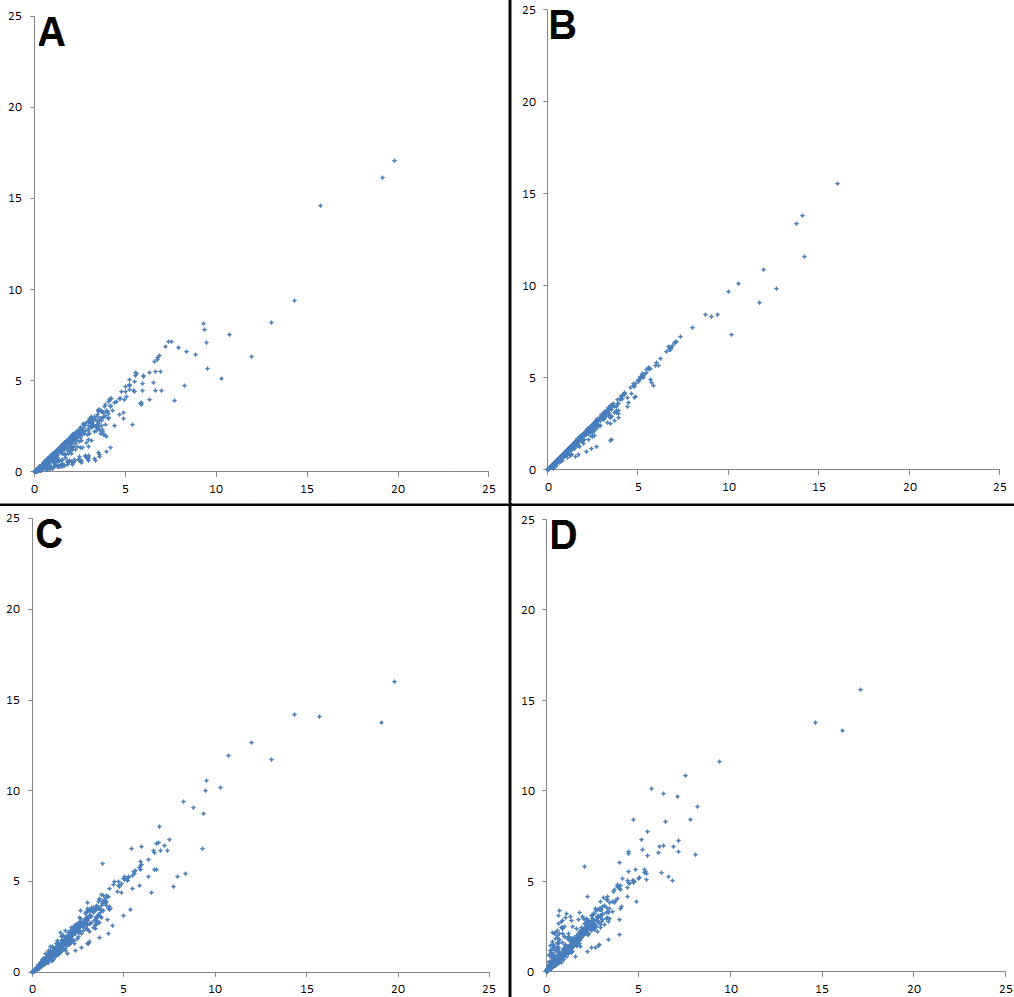
Scatter-graphs showing the effects of applying different corrections of effect size on the entire biometric data set. Scatter-graphs showing the effects of applying different corrections of effect size on the entire vocal data set. Each axis shows effect size, measured in a different way. **A** Controlling for sample size using unpooled data – *x*-axis: bare unpooled effect size; *y*-axis: controlled unpooled effect size **B** Controlling for sample size using pooled data – *x*-axis: bare pooled effect size; *y*-axis: controlled pooled effect size **C** Using pooled versus unpooled effect sizes without controlling for sample size – *x*-axis: bare unpooled effect size; *y*-axis: bare pooled effect size **D** Using pooled versus unpooled effect sizes and controlling for sample size – *x*-axis: controlled unpooled effect size; *y*-axis: controlled pooled effect size.

Tables 12 and 15 summarize the impacts of applying different tests of effect size to the 2*d* and Tobias et al. (2010) categories studies. In both the biometric and vocal studies, the least to most conservative ways of calculating effect size were: (i) bare unpooled effect sizes; (ii) bare pooled effect sizes; (iii) controlled pooled effect sizes; and finally (iv) controlled unpooled effect sizes. However, this shift was in no ways uniform, as is illustrated in Figures 2–3 and Tables 16–19.

**Table 16.** Spearman’s rank correlation coefficients as between the results of five statistical tests carried out on the vocal data set. Confidence for the values stated were given in PAST as zero or less than *p*<1x10^100^ for all tests.

Tests conducted on vocal data set	Bare Unpooled Effect Sizes	Controlled Unpooled Effect Sizes	Bare Pooled Effect Sizes	Controlled Pooled Effect Sizes
**Statistical significance (student’s *t*)**	-0.863	-0.910	-0.849	-0.859
**Bare Unpooled Effect Sizes**		0.987	0.988	0.908
**Controlled Unpooled Effect Sizes**			0.894	0.980
**Bare Pooled Effect Sizes**				0.999

**Table 17.** Spearman’s rank correlation coefficients as between the results of five statistical tests carried out on the biometric data set. Confidence for the values stated were given in PAST as zero or less than *p*<1×10^100^ for all tests.

Tests conducted on biometric data set	Bare Unpooled Effect Sizes	Controlled Unpooled Effect Sizes	Bare Pooled Effect Sizes	Controlled Pooled Effect Sizes
**Statistical significance (student’s *t*)**	-0.851	-0.951	-0.831	-0.857
**Bare Unpooled Effect Sizes**		0.936	0.990	0.983
**Controlled Unpooled Effect Sizes**			0.920	0.936
**Bare Pooled Effect Sizes**				0.994

**Table 18.** Changes in actual effect sizes resulting from changes between different methods of measuring effect sizes in the format actual mean ± standard deviation (minimum–maximum) [mean using absolute values ± standard deviation using absolute values], for voice. The figure in each cell demonstrates the outcomes of subtracting the effect size in the columns from the effect sizes in the rows, for each data point studied.

Tests conducted on vocal data set	Controlled Unpooled Effect Sizes	Bare Pooled Effect Sizes	Controlled Pooled Effect Sizes
**Bare Unpooled Effect Sizes**	0.35 ± 1.15(-0.20–24.26)[0.35 ± 1.15]	0.15 ± 0.91(-11.73–7.13)[0.36 ± 0.84]	0.24 ± 1.07(-11.32–19.28)[0.41 ± 1.02]
**Controlled Unpooled Effect Sizes**		-0.19 ± 1.55(-20.70–4.40)[0.45 ± 1.50]	-0.11 ± 1.36(-20.26–5.63)[0.40 ± 1.31]
**Bare Pooled Effect Sizes**			0.08 ± 0.46(-0.27–15.40)[0.09 ± 0.46]

**Table 19.** Changes in actual effect sizes resulting from changes between different methods of measuring effect sizes in the format actual mean ± standard deviation (minimum–maximum) [mean using absolute values ± standard deviation using absolute values] for biometrics. The figure in each cell demonstrates the outcomes of subtracting the effect size in the columns from the effect sizes in the rows, for each data point studied.

Tests conducted on biometric data set	Controlled Unpooled Effect Sizes	Bare Pooled Effect Sizes	Controlled Pooled Effect Sizes
**Bare Unpooled Effect Sizes**	0.41 ± 0.68(-0.01–5.61)[0.41 ± 0.68]	0.09 ± 0.44(-2.17–5.34)[0.19 ± 0.40]	0.21 ± 0.53(-1.97–5.77)[0.26 ± 0.51]
**Controlled Unpooled Effect Sizes**		-0.32 ± 0.73(-6.31–2.36)[0.39 ± 0.69]	-0.20 ± 0.57(-4.4–2.78)[0.30 ± 0.53]
**Bare Pooled Effect Sizes**			0.12 ± 0.28(-0.01–2.84)[0.12 ± 0.28]

The largest shift was observed between bare unpooled effect sizes versus controlled unpooled effect sizes, where a 3.9% (voice) or 9.1% (biometrics) increase in the number of outcomes in the lowest category of differentiation (0–2) was observed.

The impact of using bare pooled versus bare unpooled effect sizes is illustrated in Figures 2–3. Bare pooled effect sizes were overall more conservative than bare unpooled effect sizes. Several outcomes increased in effect size under a pooled method, which will occur where the population with the larger sample had a smaller standard deviation than the population with the smaller sample. Using pooled standard deviations generally had the result of reducing the magnitude of effect sizes compared to using unpooled standard deviations, which must relate to instances of smaller standard deviations in data sets with smaller sample sizes. This may be a natural phenomenon for highly localized and specialized populations or could result from clustering.

The overall magnitude of reduction of effect size measurements between bare pooled effect sizes and controlled pooled effect sizes was moderate. Degrees of freedom for pooled standard deviation are higher (the sum of the two samples’ sample sizes minus 2) than when using unpooled methods (where each sample is treated separately), resulting in lower *t*-values when using pooled standard deviations. Application of *t*-distribution corrections on effect sizes using unpooled standard deviations resulted in the most conservative of all measures of effect sizes, linked to overall lowest degrees of freedom in corrections and overall higher *t*-values.

Although these overall trends were observed, the impact of applying differing methods of measurement of effect sizes on actual pairwise comparisons was not uniform (see Figures 2–3). The movement to lower categories in more conservative tests was merely an overall trend, with >97% correlation according to Spearman’s ranking correlation coefficients (Tables 16–17). Even the application of “corrections” for sample size resulted in some increases in effect size measures for other sets using large samples, since *t* tends to 1.98 rather than 2 for sample sizes of over 100.

Statistical significance presented a weak negative correlation with most effect size measurements, but being most closely correlated with controlled unpooled effect sizes. In the case of biometrics, there was a strong negative correlation with controlled unpooled effect sizes (Tables 16–17). The strongest correlations were between the two effect size measurements using pooled standard deviations, which is consistent with the relatively modest correction resulting from the control for sample size, discussed above.

The variability between particular scores using different effect size measures are defined further in Tables 18–19, where positive values for the mean indicate that the test named in the column was broadly more conservative, whilst negative numbers for the mean indicate that the test named in the column was broadly less conservative. Where negative numbers are observed among the observed range of outcomes in a cell with a positive mean, this signifies cases where particular outcomes increased in measured effect size despite the application of an overall more conservative method. Up to 0.45 average magnitude of effect size change can be observed simply by applying a different method to measure effect sizes, which is a figure over double in magnitude that of the minimum effect size limit for scoring in Tobias et al. (2010)’s system. Reductions of up to 24 effect sizes magnitude were observed by controlling for sample size.

The relationship between each measurement of effect size and statistical significance is explored in Tables 20–21. Higher levels of confidence (lower values of *p*) correspond broadly to higher effect sizes in each case. However, the variation in effect size scores within each category of significance was high: some effect sizes of up to 18 were non-significant, whilst some effect sizes as low as 0.36 were significant. All scores for effect sizes falling in statistically significant categories exceeded the 0.2 limit for scoring suggested by Tobias et al. (2010), whilst many non-significant effect sizes were in excess of 0.2.

**Table 20.** Effect sizes under the four models studied here, grouped into the three “zones” of statistical significance illustrated in Figures 4–5, for vocal data, in the format: mean ± standard deviation (minimum–maximum). *p*>0.05 refers to non-significant results and corresponds to the red rhombuses in Figures 4–5. 0.5/*n_v_*<*p*<0.5 refers to possibly significant results which are excluded after applying Bonferroni correction and corresponds to the yellow squares in Figures 4–5. *p*<0.05/*n_v_* refers to statistically significant results and corresponds to the green triangles in Figures 4–5.

Statistical significance	Bare unpooled effect sizes	Bare pooled effect sizes	Controlled pooled effect sizes	Controlled unpooled effect sizes
***p*>0.05**	0.60 ± 1.31(0.00–11.56)	0.38 ± 0.33(0.00–2.21)	0.71 ± 2.13(0.00–22.92)	0.67 ± 2.02(0.00–22.47)
**0.05/*n_v_*<*p*<0.05**	1.82 ± 2.69(0.33–18.22)	1.29 ± 1.24(0.33–8.47)	1.82 ± 3.00(0.26–21.60)	1.67 ± 2.61(0.26–20.14)
***p*<0.05/*n_v_***	3.69 ± 3.30(0.36–45.33)	3.32 ± 2.73(0.37–21.07)	3.32 ± 3.05(0.37–41.45)	3.22 ± 2.81(0.37–26.05)

**Table 21.** Effect sizes under the four models studied here, grouped into the three “zones” of statistical significance illustrated in Figures 4–5, for biometric data, in the format: mean ± standard deviation (minimum–maximum). *p*>0.05 refers to non-significant results and corresponds to the red rhombuses in Figures 4–5. 0.5/*n_v_*<*p*<0.5 refers to possibly significant results which are excluded after applying Bonferroni correction and corresponds to the yellow squares in Figures 4–5. *p*<0.05/*n_v_* refers to statistically significant results and corresponds to the green triangles in Figures 4–5.

Statistical significance	Bare unpooled effect sizes	Bare pooled effect sizes	Controlled pooled effect sizes	Controlled unpooled effect sizes
***p*>0.05**	0.72 ± 0.70(0.00–3.97)	0.42 ± 0.29(0.00–1.95)	0.71 ± 0.72(0.00–3.92)	0.63 ± 0.60(0.00–3.41)
**0.05/*n_v_*<*p*<0.05**	1.45 ± 0.86(0.44–3.98)	1.06 ± 0.41(0.44–2.44)	1.37 ± 0.96(0.40–6.01)	1.26 ± 0.84(0.40–5.81)
***p*<0.05/*n_v_***	3.37 ± 2.64(0.61–19.80)	2.79 ± 2.07(0.61–17.09)	3.15 ± 2.44(0.58–16.04)	2.97 ± 2.23(0.58–15.57)

## Conclusions and discussion

The dataset studied here exhibits comparable overall levels of variation to Tobias et al. (2010)’s data set. The latter was developed using sympatric species pairs on a global basis. However, this data set involves comparisons of many populations that are currently recognized as subspecies and several of which are unnamed (Table 1). Here, 55.7% and 60.6% scored in the *minor* (score 1) category for voice and biometrics respectively (using bare pooled standard deviations), versus 58% and 63% for the Tobias et al. (2010) data set. At a score of 2 (2–5 effect sizes), this study produced scored 23.6% and 21.8% of the sample for voice and biometrics versus Tobias et al. (2010)’s 26% and 24%. This similar set of outcomes means that comparisons between outcomes of other tests are likely to be a reasonable proxy for how Tobias et al. (2010)’s database would perform, under other tests.

Several broader aspects of the results can be explained by considering the number of standard deviations’ difference required to satisfy various models (Figure 6). The lack of a control for sample size using *t*-distributions in the Tobias et al. (2010) effect size calculation resulted in a liberal approach, which may over-score differentiation at low sample sizes. However, at the 5 effect sizes level (3 points), their model low-scores differentiation for sample sizes of greater than 7 (compared to using a controlled unpooled effect size of 4) (Fig. 6). The 1-point test of Tobias et al. (2010) at 0.2 effect sizes is very liberal indeed, set at almost half the lowest recorded effect size measurement in this study that was statistically significant (0.36 effect sizes: Table 20). This inconsistency in treatment of outcomes showing very low levels of variation explains the large number of “1” scores in the Tobias et al. (2010) analysis, compared to the much smaller number of pairwise comparisons achieving Level 1 or greater variation under the Levels study.

The overall lower differentiation levels in biometrics can in part be explained due to lower sample size (see Tables 1–2) but likely also reflects lower variability of these kinds of variables.

### Pooled versus unpooled and bare versus controlled effect sizes

The outcomes of using pooled versus unpooled and bare versus controlled effect sizes are substantial across the data set as a whole and can be drastic in individual cases (Tables 18–19).

The distinction between using pooled versus unpooled standard deviations in taxonomy has passed by barely without discussion in ornithological taxonomic literature. Isler et al. (1998)’s test applies *t*-distribution data on an unpooled basis to two data sets under comparison, but Tobias et al. (2010) and Hubbs and Perlmutter (1942) used effect sizes based on Cohen’s *d* statistic, which calls for a pooled standard deviation without controlling for sample size using *t*; neither commented on their selection. Nakagawa and Cuthill (2007) recommended presenting confidence interval data and Tobias et al. (2010) refer to this, but it is unclear how this was built into their framework nor whether any cut-off based on low confidence intervals was applied.

Usage of pooled standard deviations, as a matter of statistical methodology, should only be undertaken where the standard deviations of the two populations under comparison can be assumed to be equal. This does not necessarily mean that measured standard deviations of the two populations must be equal, or even close to one another, since these will usually differ for two measured populations as a result of the sampling. However, it must be reasonable to make this assumption in order to apply this method. The pooling formula attributes greater weight to the standard deviation of the population with higher sample size and produces a “weighted average” standard deviation which is closer to that of the population of which there is a larger sample. Degrees of freedom for the pooled standard deviation are greater due to summing those of the two separate populations. In practice, in taxonomy, we will usually have no idea as to whether or not the standard deviations of two populations under comparison are equal or not. Special care should be adopted in using pooled standard deviations where estimated population sizes, molecular or geographical attributes of the two populations vary greatly. For example, comparing an isolated, very small montane population with low intra-population molecular variation versus a very widespread lowland population which is known to exhibit substantial clinal variation and has higher intra-population molecular variation would be inappropriate, since assumptions underlying the usage of pooled standard deviations are likely not just to be unknown but incorrect. The greater correlation between statistical significance and controlled unpooled effect sizes (Tables 16–17) is also noteworthy. In summary, the unpooled / Isler et al. (1998) model, which does not make unnecessary assumptions and is overall more conservative (Tables 10–15), is methodologically more supportable among the four methods for measuring effect size analyzed here.

There are still likely to be “use cases” for pooled standard deviations to measure effect sizes in taxonomy. Salaman et al. (2009) compared two populations, one being undescribed and probably extinct, known only from a single specimen. With d.f. = 0, unpooled effect size calculations produce “divide by zero” errors. In that publication, a modified version of the Isler et al. (1998) formula was developed as an indicator of diagnosis, using the standard deviation of the better-known population for both populations. In other situations, particularly those involving very small sample sizes, appropriate usage of pooled standard deviations could be considered. A particular risk when studying smaller populations with unpooled standard deviations is that sampled data points may “cluster”, resulting in small recorded standard deviations, which could exaggerate measured differentiation. Usage of pooled standard deviations can be a hedge against such outcomes, even if the underlying assumptions for using pooling are not met. However, using *t*-distributions also provides such a hedge and moreover involves a statistical test specifically designed to cater for the risk of clustering. Use case scenarios for pooled effect sizes in taxonomy are unlikely to be the norm and may not in any event justify adopting *t*-distribution-based corrections using inflated degrees of freedom.

### Statistical significance

Most papers concerning the application of statistical tests for determining the taxonomic rank of allopatric populations have noted that statistical significance is not a good measure, due to its potential for liberal satisfaction by increasing sample size, its failure to indicate higher levels of differentiation or false positives when sampling from different parts of a geographical cline; and then move quickly on to discuss better tests (Patten and Unitt 2002, Nakagawa and Cuthill 2007, Remsen 2010, Tobias et al. 2010). For example, Tobias et al. (2010) noted that: “The fact that it is easy to achieve statistically significant differences merely by increasing sample size may lead to inappropriate taxonomic decisions.” Bizarrely then, many modern taxonomic papers, including in some of the field’s most prestigious journals, erroneously claim “diagnosis” on the basis of overlapping data sets that are presented as satisfying tests of statistical significance, such as *t*-test, Tukey-Kramer, Wilks’ Lamda, ANOVA or Kruskall-Wallis tests (e.g., Benkman et al. 2009, Lara et al. 2012, Freitas et al. 2012). With this background, it is worth dwelling more on the usage of statistical significance.

Although large samples sizes are cited as the basis to reject statistical significance as a useful measure in taxonomy, such problems are rarely faced by taxonomists. Having too few specimens (whether in museums or measured in the field) or sound recordings is likely a more material problem. For new species descriptions in birds published between 1935–2009, 332 of 477 (70%) were based on 0–5 specimens, only one was based on >100 specimens and the mean number of specimens was 6 (Sangster and Luksenburg 2015). One approach to avoid false positives would be to introduce minimum criteria for sample size. Tobias et al. (2010) called, where possible, for sample sizes of at least 10. Walsh (2000) proposed that taxonomic studies using discrete data should have sample sizes in excess of 50 to exclude the possibility of polymorphisms occurring at *p* < 0.05 that cause incorrect interpretations. However, minimum limits such as these would be blunt and arbitrary and could prejudice against taxonomic recognition of highly distinct but very rare populations where only small samples are available (see also Lim et al. 2012 and Sangster and Luksenburg 2015). With continuous data, we can instead apply *t*-distribution corrections to address such concerns.

The classic test of statistical significance between two populations of data is the Student’s *t*-test. This evaluates the probability of whether two normally-distributed data sets relate to two different populations, by considering whether or not their mean averages are likely to differ from one another. Various other similar tests can assess differences between mean, median, or modal averages, such as *F*, Mann-Whitney *U*, Kolmorov-Smirnov, Wilks’ Lamda, ANOVA, Kruskal-Wallis, and Tukey-Kramer. Some of these tests are better suited to continuous variables which are non-normally distributed, such as ratios or products of raw data.

Although the *t*-test will evaluate the likelihood that two populations are different, it tells us little about the extent of differences between the two populations. With a large enough sample, the two sample means may be very close to one another. Here, the lowest distance between statistically significant outcomes was 0.36 effect sizes. Tests of statistical significance can also be failed on data showing effects sizes as high as 18 (Table 20), where sample sizes are small. An example of data with close means passing a test is the following, based on a large sample with almost complete overlap of variables:

Donegan (2012, Appendix 3A and Appendix 4): Maximum acoustic frequency of last note of male song (kHz). Data are in the form *average ± SD (lower bound – upper bound) (n = sample size)*:

*Myrmeciza melanoceps*: *2.42 ± 0.12 (1.98–2.62) (n = 143)*

*Myrmeciza goeldii*: *2.29 ± 0.08 (2.01–2.49) (n = 173)*

The *t*-test was passed at *p*<0.0002, yet these data reveal small differences between means and substantial overlaps in recorded values. The *t*-test result suggests that the two populations in question have begun to diverge from one another, which is interesting and makes it valid to discuss their relationship and possible isolation mechanisms. However, identification of a sound recording to one or the other species on the basis of these data would be impossible. The effect size here was 1.31, considerably in excess of the lowest (0.36) score, but fewer than 50% of individuals could be identified based on this variable and it would be useless for identification. Regularly, diagnosis is incorrectly asserted in the taxonomic literature based on data like those in this example (see citations above).

The *t*-test and similar tests demonstrate *statistical significance* of differences between means. Such differences may have some evolutionary significance. However, a positive *t*-test is not necessarily of much *taxonomic significance* (Fig. 1): we must also consider how much the two populations have differentiated.

In the field of medicine, the outcome of tests of statistical significance is widely understood and accepted to be just a first phase in demonstrating an interesting result. A variety of different approaches exist in medical science which must also be passed to show *clinical significance*, which, for example, would support the usage of drugs. In any taxonomic study, it is similarly important to move on from the ecology class, beyond *statistical significance* to consider the *taxonomic significance* of any results.

**Figure 4.**
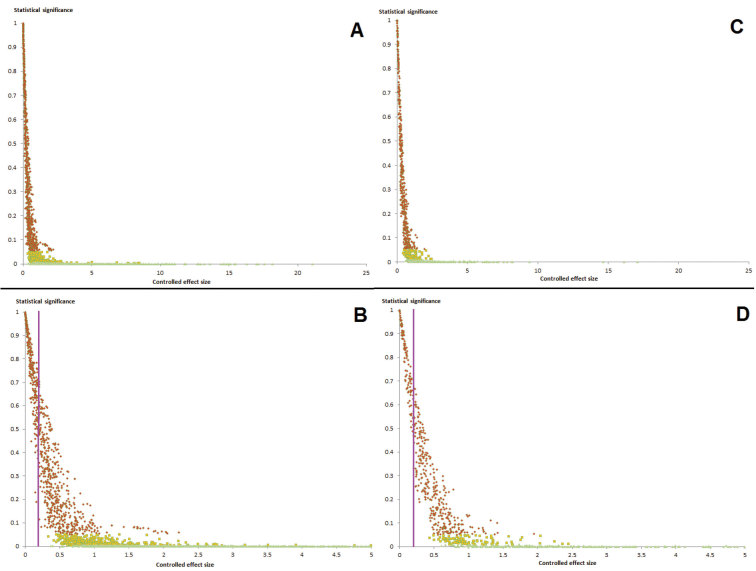
Scatter-graphs of controlled unpooled effect size (*x*-axis) versus statistical significance (*p*<x) (*y*-axis). All outcomes to the right of the two purple lines represent pairwise comparisons which are given scores of at least 1 under the Tobias et al. (2010) system. Note the statistically insignificant outcomes (red rhombuses, to the right of the purple line) which are given scores of 1 under the Tobias et al. (2010) system and the small numbers of such outcomes scoring 2 (two effect sizes or more) and 3 (five effect sizes or more). Figure 4A, B use all 2348 pairwise comparisons for voice. Figure 4C, D show all 822 pairwise comparisons for biometrics. Figure 4B, D are close-ups of Figure 4A, C respectively, showing the area below 5 effect sizes. Red rhombuses represent a lack of statistical significance. Yellow squares represent possible statistical significance, less than *p*<0.05 but failing tests of significance after applying Bonferroni correction. Green triangles, which are almost continuous along the *x*-axis, represent statistically significant outcomes. The lower two graphs show greater resolution of the same graphs at effect sizes of up to 5 (where 4 signifies full diagnosis). Note: the graphical representation is generous to Tobias et al. (2010) in applying the most conservative effect size definition from this study on the *x*-axis; using bare pooled effect sizes as applied by these actions would generally shift outcomes to the right, as illustrated in Figures 2–3.

That all said, statistical significance can be a tougher one than some proposed measures of differentiation. Instances were found here of pairwise comparisons passing Isler et al. (1998)’s gold standard of diagnosability but failing tests of statistical significance (Table 6: 15 outcomes, or 0.6%, in categories 2, 3, 4 & 5 or 2, 4 & 5). Instances of differentiation being scored under the Tobias et al. (2010) system in statistically insignificant situations were widespread (Figure 4). Under the Tobias et al. (2010) system, up to 87–91% of outcomes studied here scored 1 or more points (Tables 13–14) but on the same data set 49.5% (voice) to 64.6% (biometrics) of outcomes failed tests of statistical significance (Tables 8–9), suggesting false attribution of scores to non-significant situations under this system in at least 36% of cases (see Figure 4). This present study therefore highlights the importance of considering both significance and effects-based differentiation in taxonomy. Demonstrating that the means of two populations have actually diverged at all (using statistical significance) should be a baseline requirement for any assertion of diagnosis between two populations, an omission in both Isler et al. (1998)’s and Tobias et al. (2010)’s systems. To avoid “false positive” assessments of diagnosability, the *t*-test or another test of statistical significance between means should be introduced as a gateway and *additional* requirement to tests of diagnosis. It also flows from this study (Figure 4, Tables 20–21) that an effect size of 0.2 cannot be supported as a basis for attributing taxonomic significance when using continuous variables.

### Type 1 error corrections

Introducing the Dunn-Šidák correction (as opposed to the simpler but overly conservative Bonferroni correction) had a virtually negligible effect (Tables 4–5). As a result, for taxonomic data sets involving similar numbers of variables to those addressed here (fewer than 30), it will probably not be worth the trouble of applying more complex corrections.

Bonferroni (and Dunn-Šidák) corrections are appropriately applied to “families” of variables. A middle-ground of treating voice and biometrics as separate “families” is recommended based on this study. This could be criticized, since certain aspects of voice and biometrics can be linked (e.g., Podos et al. 2004, Phillips and Derryberry 2017). However, Bonferroni is inherently conservative: for example, biometric measures are likely to be correlated with one another and so may not be truly independent: for example, longer-winged birds might be more likely to have longer tails as well, due to environmental or hereditary conditions affecting feather growth or size. The proposed liberalism here of treating voice separately from biometrics counterbalances the over-compensation inherent in Bonferroni between data sets in variables that may show some correlations. Treating these variable families separately can be justified since many taxonomic studies (including some included here) address only one or the other kind of variables. Applying statistical corrections based on the full number of variables studied makes results less comparable and penalizes more holistic studies. In contrast, it would seem less justifiable to apply a more liberal standard to, say, one type of call versus another type of call. It was found when re-analyzing these data that such an approach resulted in a more liberal approach to “less variable-rich” vocalizations, such as single-note calls. This seems inappropriate given that such calls are likely less relevant to mate choice than male songs, which tend to be more complex and require more variables to properly analyze. (This proposed family-treatment of variables based on all vocalization-types for purposes of Bonferroni correction presents a change from the methodology underlying several of the studies listed in Table 1.)

### Diagnosability

Diagnosability was considered to be the most frequently applied criterion to assess rank in a review of over 1000 taxonomic revisions (Sangster 2014). It is an important concept, not least because the International Code of Zoological Nomenclature (ICZN 1999) recommends that: “When describing a new nominal taxon, an author should make clear his or her purpose to differentiate the taxon by including with it a diagnosis, that is to say, a summary of the characters that differentiate the new nominal taxon from related or similar taxa” (Recommendation 13A). Under Article 13.1.1 of the Code, a newly described species name is not “available” unless it is “accompanied by a description or definition that states in words characters that are purported to differentiate the taxon” (or includes reference to a text which does so). Moreover, diagnosability allows for identification, which enables users of names to label populations.

Dubois (2017) has provided some interesting insights into diagnosability as a concept in taxonomy and nomenclature. However, he did not directly address its measurement and statistical evaluation using data sets based on continuous variables, which are the focus of this paper. The two models analyzed here that address diagnosability are: (i) that of Isler et al. (1998), which is based on controlled unpooled effect sizes of 4; and (ii) that of Tobias et al. (2010) which attributes a score of 3 to situations where the means differ by 5 bare pooled effect sizes. There are clear advantages to controlling for sample size in assessing whether any overlap exists, in that diagnosability tests would then not bias against studies using different sample sizes. The Isler et al. (1998) test also benefits from a statistical and conceptual purity in measuring a meaningful mathematical and statistical situation of diagnosis to 95%, compared to an arbitrary number of 5 effect sizes which often exceeds what is needed to demonstrate statistical diagnosis.

In this study, 14.5% of the vocal data set and 6.3% of the biometric data set passed the Isler et al. (1998) diagnosability test (“Level 5”, Tables 8–9). Tobias et al. (2010)’s equivalent test of “5 effect sizes” (including scores of over 10) was met by fewer population/variable comparisons for voice, but more comparisons for biometrics: 10.1% and 7.4% respectively (Tables 13–14, data for bare pooled standard deviations). It is expected that an effect size of 5 would result in fewer positive outcomes than an effect size of 4. The larger number of positive outcomes for biometrics at 5 effect sizes is due to using pooled standard deviations and consequent impact of controlling for sample sizes using lower values of *t.* A rationale for excluding the 4.4% of tests in the vocal sample which met a 4 standard deviations standard (controlled for sample size) but failed a test of 5 bare pooled standard deviations as diagnosably distinct is elusive. It would be prudent for the 3 point score of Tobias et al. (2010) to be recast so as to align to the Isler et al. (1998) test (as modified here), as was done in Donegan and Avendaño (2014). Tobias et al. (2010) adopted a model which is a conservative proxy for diagnosis and simple to calculate. However, whilst overly liberal in the trigger for assigning 1 point, they were unnecessarily conservative in setting a higher trigger for assigning 3 points.

### Differentiation below diagnosability, subspecies, and the 50% and 75% tests

There is no consensus as to whether any differentiation below diagnosability for particular characters ought to be recognized in taxonomy. Remsen (2010) essentially rejected this proposition, requiring a valid subspecies to show 95% diagnosability in “one or more phenotypic traits” and “not to multiple simultaneous comparisons”. This can be equated to a single character meeting the Isler et al. (1998) standard of >4 controlled standard deviations in a single character. There are however conceptual difficulties with models that depend on diagnosability of a single character. Two taxa may show considerable overlap for two or more variables if these characters are analyzed separately, but may be diagnosable in multivariate space. Quantitative criteria based on a series of univariate datasets are liable to overlook significant diagnosis below full differentiation and could result in taxonomic non-recognition of diagnosable populations. Elucidating diagnosable characters is important to satisfy the requirements of the ICZN code and for identification, but any morphological or vocal character identified by taxonomists is simply a measurable slice of multivariate character space differentiating two populations.

Originally, 75% diagnosis tests for subspecies used one of two approaches: (i) a 75%/75% test (i.e., 75% of population 1 is diagnosable from 75% of population 2; or (ii) a 75%/99+% test (i.e., 75% of population 1 is diagnosable from essentially all of population 2). Amadon (1949) opined that any measure below 75%/99%+ diagnosability “does not seem to set a high enough standard”. Patten and Unitt (2002) also reaffirmed use of this measure, but added to Hubbs and Perlmutter (1942) and Amadon (1947)’s framework by controlling for sample size using *t*-distributions, based on a similar method of adjustment to that of Isler et al. (1998). They noted that, where this 75% method is applied controlling for sample sizes, it achieves outcomes close to those obtained for full diagnosability, as also commented by Remsen (2010). Hubbs & Perlmutter (1942)’s conceptual alternative of a 50%/100% test was effectively adopted in part as a benchmark by Tobias et al. (2010) by giving two points in their scoring system to populations which are over 2 bare pooled standard deviations apart.

The application of sharp, seemingly arbitrary, tests such as these to classify normally distributed data into segments to which scores are attributed is a situation not unique to taxonomy. Similar hard boundaries are also rife in most education and examination systems. In UK universities, a student scoring 60.1% or 69.9% in an examination will be given the same award (an upper second class degree) but a student attaining 70.1% will get a different award, a first class degree. This is despite the students scoring 69.9% and 70.1% having attained more similar levels of achievement to one another. Whilst any cut-off may be criticized as arbitrarily generous or harsh to outcomes falling close to the line on either side, the application of cut-offs is something that humans tend to do in their quest to categorize things. Where cut-offs are applied, a test of whether the cut-off is a valid one should best be based upon: (a) differentiation of a meaningful number of outcomes; and (b) the setting of boundaries at statistically-, mathematically-, or biologically-meaningful positions.

In this data set, with very large numbers of pairwise comparisons, necessarily many individual cases fall very close to each of the cut-off boundaries proposed by previous models for attributing taxonomic significance, whether at or below diagnosability. Two populations differing by 95% using Isler et al. (1998)’s diagnosis test will be given credit for that character, but two populations differing by 94.9% will not. However, this seemingly arbitrary distinction is supportable because 95% is the standard confidence interval used in science. In contrast, the 75% test of subspecies flunks both requirements for a supportable test of differentiation. Depending on the sample size, the 99/75% concept equates to an average diagnosability of over 90%+ per population (Patten and Unitt 2002, Remsen 2010), and so gets close to a species test. Because *t* at 99% can exceed *t* at 97.5% with very small samples, it is even sometimes the case that *t* at 99% + *t* at 75% exceeds 2*t* at 97.5%, explaining the 2.3% of outcomes in Table 6 of pairwise comparisons passing the Level 5 test of 95% diagnosis but counterintuitively failing the Level 3 test of 75% diagnosis (Levels 1245 or 125 therein). In this study, the 75% category was narrow to the extent of being almost negligible for both vocal (2.2% of sample) and biometric (1.2%) data (Tables 8–9). Neither does 75% differentiate any biologically meaningful or statistically meaningful delineation of which I am aware. It merely provides a marginally more liberal subspecies test than that proposed by Remsen et al. (2010) for most sample sizes. This 75% test should be abandoned altogether, as was also proposed by Remsen (2010).

In contrast to the 75% (Level 3) test, 50%/95% differentiation (Level 2) measures a mathematically relevant point of differentiation, when the mean of one population moves outside the normal distribution of the other. It also signifies the point at which a population has moved half way towards diagnosability. The number of pairwise comparisons meeting the Level 2 test (but not falling in other buckets) was material but not enormous. Only 30.6% for voice and 20.6% for biometrics of outcomes passed this test at all (Tables 8–9). Its outcomes compare to Tobias et al. (2010)’s scoring of over 70% of outcomes and the number of outcomes meeting tests of statistical significance. Only 13.9% and 12.9% of the sample respectively, for voice and biometrics, fell into a category where the 50% Level 2 test (but not others) were met, totals which rise to 16.1% and 14.1% respectively if the results of the (broadly useless) 75% category are aggregated. Once sample size is controlled for using *t*-distributions, 50% differentiation arguably becomes the closest defendable proxy to the traditional 75% test of subspecies on which most avian taxonomy was built, if one takes into account the example studies of Hubbs and Perlmutter (1942) and Amadon (1949) and the sample sizes that they used. When controlling for sample size, a 50% test can be quite an exacting standard to pass and gives considerable comfort that material differentiation has taken place. In contrast, adopting a 75%/99%+ diagnosis (near-diagnosability) as a test for subspecies rank would place many currently recognized and largely identifiable geographic variants of birds occurring on different mountain ranges into synonymy, which is undesirable because many of these populations have been shown to merit taxonomic recognition based on studies of plumage or molecular characters. The most extreme example of low differentiation calling for taxonomic recognition in the study here relates to the Yariguíes and northern East Andes population of Speckled Hummingbird *Adelomyia melanogenys*, which reached only up to “Level 2” differentiation in voice, no differentiation in biometrics and are near-diagnosable (but non-diagnosable) in plumage. However, they exhibit *c.*5.8% mtDNA differention (Chaves and Smith 2011). Such populations should arguably be recognized taxonomically, at least as subspecies.

### Diagnosis based on actual data

In addition to the diagnosis formula for Level 5, Isler et al. (1998) require satisfaction of the Level 4 test of non-overlap. Although such considerations can help identify situations requiring further investigation, the Level 4 test biases towards positive outcomes in studies using small samples (Fig. 6). For pairwise comparisons which narrowly meet Isler et al. (1998)’s 95% diagnosis test, it would be expected for 5 sampled measures out of 100 actually to overlap. For such a data set, satisfaction of the Level 4 test could be random in that a *p*<0.05 result might arise at any point of data collection between *n* = 1 and *n* = 100, rendering Level 4 unsatisfied and denying credit for observed differentiation. The likelihood of an outlier existing in the sample increases on a linear basis with sample size. There were 22 instances in the vocal study (0.9% of outcomes) most of which included populations with *n* > 100 sample size, in which levels 1, 2, 3 & 5 were passed (Table 6), i.e., all statistical tests were passed except actual non-overlap. A rationale for denying significance to these outcomes is elusive, but retaining this criterion penalizes studies using large sample sizes. Usage of this test as a gateway to affording weighting to observed differentiation in taxonomy should be abandoned.

### Adapting Tobias et al. (2010)

Tobias et al. (2010)’s standard of 0.2 effect sizes as a starting point for attributing taxonomic significance was probably based upon Cohen (1998)’s original scheme for interpreting effect sizes, as embellished by Sawilowsky (2009), in which effect sizes are categorized as “small” above 0.2, “medium” above 0.5, “large” above 0.8, “very large” above 1.2 and “huge” above 2. This study shows the supposedly unusual “huge” category actually to be fairly standard in taxonomy, with 33–37% of vocal comparisons achieving this benchmark (see further the discussion above on 50% differentiation). Isler et al. (1998) and Remsen (2010) only value effect sizes of 4 (double, “huge”) and no less. Here, the highest effect size recorded between relevant taxa, all of which were considered congeners at the time of the study, was over 40. Effect sizes of over 10 represented over 3% of the vocal sample. Tobias et al. (2010)’s higher scores also attribute second and third points at 2 (“huge”) and 5 (more than double “huge”), such that some acknowledgement of the inappropriateness of traditional effect size interpretations is evident in their system.

Traditional interpretations of effect sizes may be appropriately used in other fields but are inappropriate for taxonomic study. It should be borne in mind that the traditional subjective descriptors for effect sizes starting at 0.2 have been developed largely in the fields of social and behavioral science (Cohen 1998, Sawilowsky 2003). Such fields by definition only consider intra-specific differences (typically, within *Homo sapiens*) and not between-species differences. In taxonomy, we are primarily interested in diagnosability and identification, which look to greater levels of differentiation.

Overall, this study suggests that: (i) Tobias et al. (2010)’s score of 1 for minor differences is set at too liberal a level, attributing taxonomic value to differences which in many situations are of no statistical significance; (ii) their scores of 1, 2, 3, and 4 are all based on bare pooled effect sizes, which involve a certain degree of “hedging” against measured standard deviation error, but do not include a sufficient control for sample size and are based on inappropriate assumptions; (iii) the score of 3 is based on 5 standard deviations’ difference, which is an arbitrary value set unnecessarily high when 4 effect sizes equates to diagnosability, and which is overly conservative for any study with a sample size greater than 7 but overly liberal otherwise (Fig. 6); (iv) the score of 4 is based on 10 SDs’ difference, a very high standard to meet for any data set, and also set arbitrarily; (v) total measured variation in effect sizes may theoretically vary by a factor of up to 3, for different situations which attain the species benchmark score of 7 points (Table 25); and (vi) the overall scheme results in a homogeneous scoring system where almost every comparison attains 1–2 points and few comparisons get no or higher scores. Several challenges arise in recalibrating the model. First, the only supportable measures below diagnosability studied here are considered those of statistical significance (Level 1) and 50% diagnosability (Level 2), yet the Tobias et al. (2010) system calls for two scores (1 and 2) below the level of diagnosability and a score of 3 which seems intended to approximate to diagnosis. Secondly, the comparison with sympatric “good” species in Tobias et al. (2010) would need to be re-run entirely to check whether the benchmark score of 7 for species rank requires modifying if their model is modified. I propose here two possible approaches which should be explored further to improve the scoring system’s application to continuous variables:

Solution A:

1 point: Level 1 statistical significance only.

2 points: Level 1 plus Level 2 50% diagnosability.

3 points: Level 1 plus Level 5 full diagnosability (3 points).

4 points: Level 1 plus a new measure of a “species and a half” worth of diagnosability (equivalent to 6 controlled effect sizes).

Solution B: would use more proportionate scoring, eliminate the weighting for statistical significance and allow only three scores:

1.5 points: Level 1 plus Level 2 (2 controlled effect sizes).

3 points: Level 1 plus Level 5 (4 controlled effect sizes).

4.5 points: Level 1 plus 6 controlled effect sizes.

Solution C: would abandon these various cut-offs and instead use controlled unpooled effect sizes, calibrated by a scale factor such that no difference = 0 and full diagnosability = 3 and capped at a score of 4.

As has been argued elsewhere (e.g., Remsen 2015, Donegan et al. 2015), the Tobias et al. (2010) system should be restricted to situations of allopatry and their positive scorings for hybridization should be removed from the model. Despite the above conclusions, Tobias et al. (2010)’s proposals represent an important step forwards towards an holistic measure of species rank (incorporating plumage, habitat, voice and biometrics data) and so have several notable benefits and important objectives in light of rationality issues affecting modern taxonomy. This holistic approach also has benefits over systems which consider only continuous data. It also seems that Del Hoyo and Collar (2014, 2016) in practice did not attribute vocal and biometric scores to situations of trifling differentiation and, as a result, the recommendations in those works are likely to be more closely aligned to outcomes under the amended basis for attributing scores discussed above.

### Amendments to the Isler et al. (1998) method

For the reasons above, the Level 4 non-overlap test should be abandoned from this framework in order to positively score the 2.5% of vocal outcomes which were diagnosable to 95% but actually overlapped due to very large sample sizes (Table 6). This 2.5% of overall outcomes represented 16.4% of those with positive “Level 5” tests. Secondly, statistical significance using a *t*-test should be assessed as a gateway to concluding any positive outcome of diagnosability, in order to avoid counting false positives. These made up 0.6% of overall outcomes including 4% of outcomes with positive “Level 5” tests. These amendments are lower in their impact compared to those proposed here to the Tobias et al. (2010) system.

**Figure 5.**
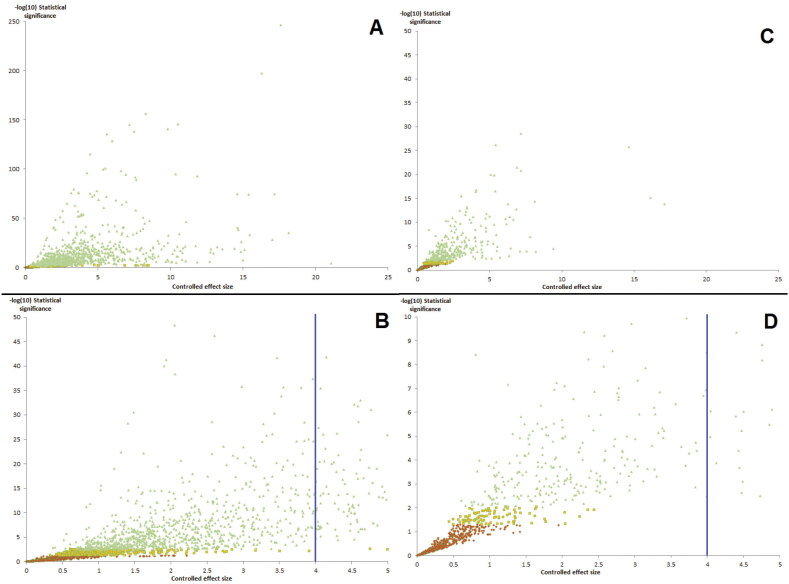
Logarithmic plot of the same data as in Figure 4, showing controlled unpooled effect size (*x*-axis) versus a logarithm of statistical significance (*p*<x) (*y*-axis). Note the number of instances of statistically significant variation ignored under the Isler et al. (1998) approach (green triangles, to the left of the purple line). Figures 5A, B use all 2348 pairwise comparisons for voice. Figure 5C, D show all 822 pairwise comparisons for biometrics. Figures 5B, D are close-ups of Figures 5A, C respectively, showing the area below 5 effect sizes. Red rhombuses, which are almost continuous along the *x*-axis on the left hand side of the two upper graphs, represent failure of tests of statistical significance. Yellow squares represent possible statistical significance, less than *p*<0.05 but failing tests of significance after applying Bonferroni correction. Green triangles represent statistically significant outcomes. Those outcomes to the right of the purple line are given credit as diagnosable under the Isler et al. (1998) test of species rank. Note also in the upper graphs the handful of yellow squares at effect sizes of greater than 4, which represent statistically insignificant outcomes which are nonetheless given credit under the Isler et al. (1998) model.

A disadvantage of the Isler et al. (1998) method remains its general exclusion from consideration of situations which were statistically significant but non-diagnosable, making for conservative interpretations. However, conservative interpretations are conceptually less supportable than interpretations based on a “best view” of available data, give precedence to history or tradition over rationality, reinforce geographical biases in status quo taxonomies and ultimately misinform biodiversity conservation and other users. I have been particularly frustrated in the past at being required by peer reviewers at the same time to (i) show satisfaction of all species or subspecies concepts in order to describe or recognize a new or synonymized taxon, but (ii) at the same time being asked to disprove satisfaction of all recognized concepts in order to lump a taxon (e.g., see Donegan and Avendaño 2008, 2010). The result of such requirements is the non-description of equally diagnosable taxa as those recognized in current taxonomies or the non-lumping of presently recognized but dubious taxa. Moreover, as illustrated in Figure 5, outcomes from comparisons showing differentiation below diagnosability represented a rich source of potentially useful information, which can be taken into account using other methods developed below.

### “Hard cut-offs” in existing models of species rank and their elimination

Isler et al. (1998) and Tobias et al. (2010)’s models both suffer from a common shortcoming. Testing whether two allopatric populations are or are not as differentiated as two sympatric species (Helbig et al. 2002) will mean that, in a large data set, some will just pass and some will just fail. However, a difficulty embedded in existing systems of assessing rank is that they create a series of further examinations, all of which have their own inflection points, in order to come to an answer (Figure 7).

**Figure 6.**
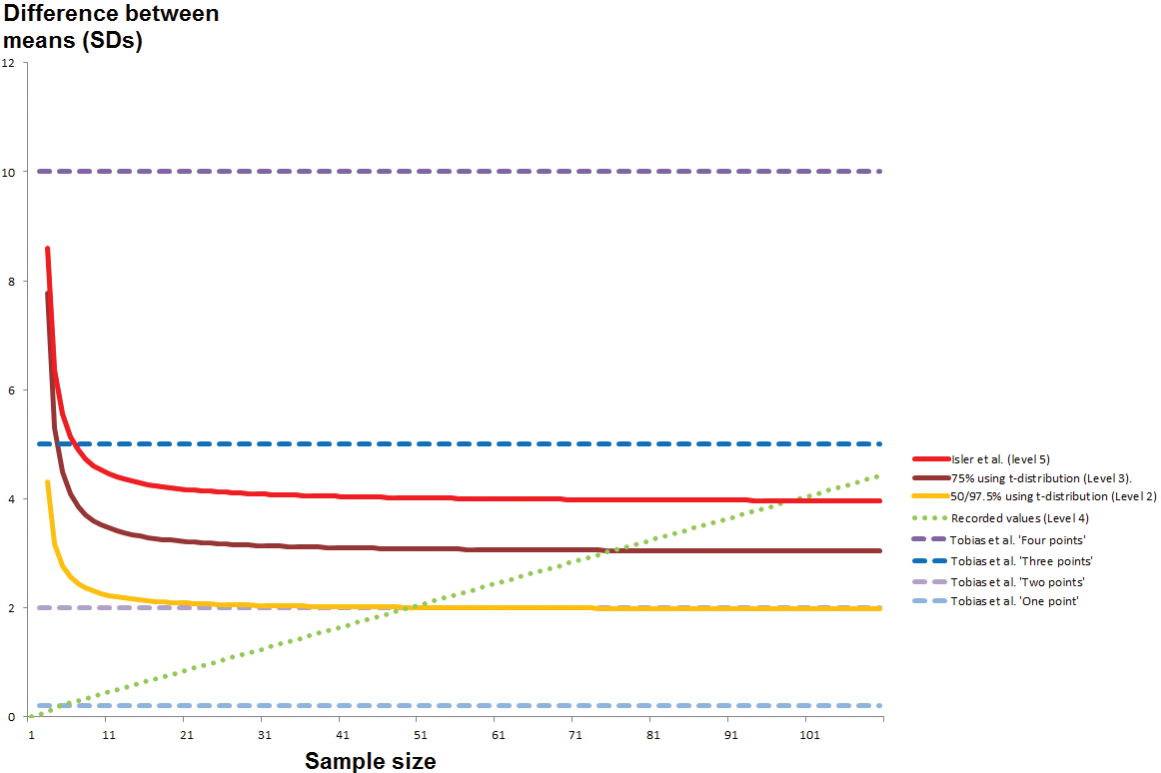
Graph showing relationship between sample size (*x*-axis) and numbers of effective SD differences between means or effect sizes (*y*-axis) required in order to pass a test of diagnosability shown in the legend. Dashed lines represent the four boundaries for affording scores under Tobias et al. (2010). Solid lines represent the Levels 2, 4 and 5 (Isler et al. 1998) tests of diagnosis. The dotted line is based on diagnosability using actual values, for a pairwise comparison of two populations which marginally meet the Level 5 test, where results falling outside a 95% distribution are averaged out in their linear occurrence in the data set. In reality, a data point outside of the 95% distribution could occur randomly at any point along this line, including as the first data point or as data point numbers 96–100. Differences arising from usage of unpooled versus pooled standard deviations are ignored for purposes of simplicity.

**Figure 7.**
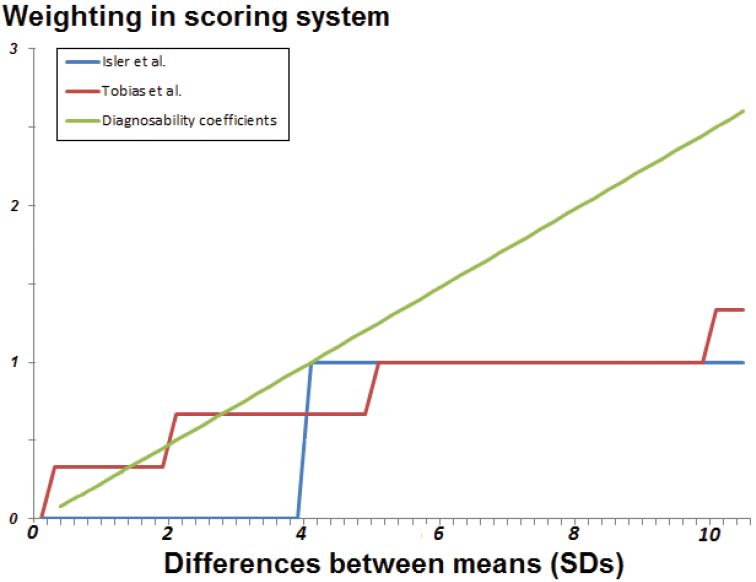
Graph illustrating the “hard cut-off” approaches of Isler et al. (1998) and Tobias et al. (2010). The *y*-axis shows the score attributed under the relevant system, weighted for 1 = diagnosability. The *x*-axis shows effect sizes. In addition to division by three, the Tobias et al. (2010) scores are treated more conservatively by assuming that bare pooled effect sizes are equivalent to controlled unpooled effect sizes. The scores for effect sizes at the lower end of the graph are somewhat artificial with a starting score of 0.34. This is based on the lowest recorded controlled unpooled effect size which passed a statistical significance test for biometrics (see Table 20). In reality, some lower differentiation with larger samples will be scored and some higher variation with lower samples will not be scored at all: see Tables 20–21.

One could adapt the Isler et al. (1998) test into a universal test, as follows (using the definitions set out in the formula in the next following section):

∑[*n*=1 IF (min *s*_1_>max *s*_2_ OR min *s*_2_>max *s*_1_) AND (|(*x̄*_1_–*x̄*_2_)| > *s*_1_(*t*_1 @ 97.5%_) + *s*_2_(*t*_2 @ 97.5%_))]

≤

∑[*n*=1 IF (min *s*_3_>max *s*_4_ OR min *s*_3_>max *s*_4_) AND (|(​​*x̄*_3_–*x̄*_4_)| > *s*_3_(*t*_3 @ 97.5%_) + *s*_4_(*t*_4 @ 97.5%_))].

Such a hard-edged statistical framework would go beyond the recommendations of Isler et al. (1998) test, who presented their method as a “point of reference” and not a requirement, and take other considerations such as plumage and not-quite-diagnosable characters into account. In practice, those using this method also identify characters that barely failed the test or for which there was an outlier that may cause overlap and consider morphological and other evidence that might be relevant to species recognition (M. Isler in litt. 2016). However, this is only so necessary because the statistical method itself suffers from a shortcoming. A drawback of the system, if applied rigidly, is that those pairs which pass two vocal diagnosability tests very easily will fail to meet the requirement of species rank, even if a third, fourth, fifth and sixth vocal variable fail the test by only a tiny margin.

The Tobias et al. (2010) test is less severely impacted by cut-offs (Figure 7) because it takes data from a broader variety of sources and partitions scores into different marks of up to 4, rather than applying a single cut-off scored on a 0/1 basis. This is in principle a step forwards compared to the Isler et al. (1998) model. However, the Tobias et al. (2010) model still uses hard cut-offs.

There is a relatively simple solution to this shortcoming: with continuous data, to move away from models which attribute cut-offs and instead to apply precise scoring under a system which only uses a hard cut-off at the very final point of determining species or subspecies rank. Such an approach was effectively attempted in a recent study (e.g., Freeman and Montgomery 2017 discussed below) and is also applied through methods such as multivariate statistics. However, multivariate techniques fail to illustrate the full range of variation in multidimensional space, do not test particular characters for diagnosability, and are often presented in a way that affords little assistance to identification. Multivariate tests also require a “complete” set of variables for each individual data “row” which precludes applying the technique to an holistic set of data based, for example, on both in-the-field sound recordings and museum specimen measurements of the same population.

### Note on Freeman and Montgomery (2017)

Immediately prior to going to press, Freeman and Montgomery (2017) compared measured differentiation in voice between pairs of allopatric birds against their own bespoke measures of responses to playback using field studies. They measured differentiation by analyzing bare pooled effect sizes, applying a conversion to standardize the data set for a universal mean of 0 and standard deviation of 1, ran principal component analysis of the modified data set and then took the measure on the PC1 axis as a surrogate for between-population differentiation. The PC1 axis was found to measure 48% of observed variation in multidimensional space. This method shares a number of close parallels with the methods that will be set out in the next section, in that it attempts to take all measured variation into account and avoids the usage of any hard cut-offs or scoring system except at the point of final diagnosis. Their conversion to SD of uniformly 1 has the same result as the effect size measures used here. Their method does however share several non-optimal aspects of previous studies, in particular: (i) failing to exclude statistically insignificant comparisons; (ii) using bare pooled effect sizes, when controlled unpooled effect sizes are recommended here; and (iii) discarding 52% of observed variation at the last stage, by relying on a single principal component value. Freeman and Montgomery (2017)’s method could be improved in variation capture by measuring centroid distance between PC1 and PC2 and eliminating non-significant outcomes. Other drawbacks of multivariate methods referred to above apply equally here. These authors highlight the importance of playback studies to assess the “allopatric problem” in birds. Considering the results of the analyses proposed here together with the results of molecular studies, playback studies and studies of discrete characters in an holistic manner is of course important in coming to a more informed view of particular taxonomic questions.

### A new universal system for measuring differentiation

In this and the next section, a new, universal measure of differentiation is developed. It is potentially usable in any taxonomic group where continuous variables are studied and in other contexts to measure effect sizes.

Step 1: identify a comparison group.

For an assessment of the rank of allopatric populations, this method compares: (i) two sympatric and closely related populations which are demonstrably good species and broadly accepted as such (Species 1 and Species 2) as well as (ii) two allopatric populations under study (Population 3 and Population 4). Ideally, Species 1 and Species 2 should also be sister taxa or be known or suspected to be very closely related through molecular studies, such that they represent a good benchmark. However, this may not always be known for certain. Preferably, Species 1 and 2 and Populations 3 and 4 should all be congeneric, but this might not be possible and they might be merely a good example from the same family or order, depending on how speciose the relevant higher-level taxonomy is. Either (but not both) of Population 3 or Population 4 might be the same as Species 1 or Species 2 or they may all be different populations.

Step 2: collect data for relevant variables using continuous measurements.

It is critical to ensure a fair identification of variables, which adequately and honestly document the maximum possible observed variations between all populations (i.e., not just the allopatric pair, but also the sympatric pair). Variables differentiating sympatric Species 1 and 2 should not be overlooked, even if more time is spent studying allopatric Populations 3 and 4. Returning to the theme of taxonomic significance and not simply statistical significance, it is important that the variables under study are likely to be taxonomically relevant. Field experience or knowledge of the organisms concerned is important to avoid splits or lumps being published based on statistical tests applied to inappropriately selected variables.

Unlike in multivariate statistics, the technique presented here will not require each data set to have the same measures from the same individuals. This means that a biometric data set based on museum specimens and a vocal data set based on a different set of individuals and with different sampling can be combined, so data from all possible sources can be collated and combined. The broadest possible geographical and numerical sampling is important (e.g., Isler et al. 1998, Tobias et al. 2010).

Step 3: undertake pairwise comparisons using controlled unpooled effect sizes.

The following formula should be applied to measure controlled unpooled effect sizes on a pairwise basis, separately for each population/variable combination under study, e.g., for Species 1 and Species 2:

|(​​*x̄*_1_–*x̄*_2_)| / ¼[*s*_1_(*t*_1 @ 97.5%_) + *s*_2_(*t*_2 @ 97.5%_)]

Step 4: exclude all the statistically insignificant data.

Comparisons showing no statistical significance should be eliminated and scored as 0. This process needs conducting separately for each population/variable combination under study: a variable might be scored as zero as between Species 1 and Species 2, but may be scored positively as between Population 3 and Population 4. Bonferroni correction is applied here, in order to keep the formula simple and due to the near-nil impact of using less conservative “type 1” error corrections. It is recommended that different sets or “families” of data (biometric, vocal, colorimetric) are treated separately for purposes of determining the appropriate Bonferroni correction. Other more complex “type 1 error” corrections such as Dunn-Šidák should be considered for situations where very large numbers of variables are compared. The exclusion of statistically insignificant data results in the following modification to the effect size formula above, e.g., for Species 1 and Species 2:

*p*<0.05/*n*_v_ → |(*x̄*_1_–*x̄*_2_)| / ¼[*s*_1_(*t*_1 @ 97.5%_) + *s*_2_(*t*_2 @ 97.5%_)]

Step 5: add up all the results of the above calculations (using a Euclidian approach).

It would be simple then to add up all the effect sizes, as follows, and see whether Species 1 vs Species 2 or Population 3 vs Population 4 had the better score. This would apply the formula:

∑ [*p*<0.05/*n*_v_ → |(*x̄*_1_–*x̄*_2_)| / ¼[*s*_1_(*t*_1 @ 97.5%_) + *s*_2_(*t*_2 @ 97.5%_)]]

≤

∑ [*p*<0.05/*n*_v_ → |(*x̄*_1_–*x̄*_2_)| / ¼[*s*_3_(*t*_3 @ 97.5%_) + *s*_4_(*t*_4 @ 97.5%_)]]

**Figure 8.**
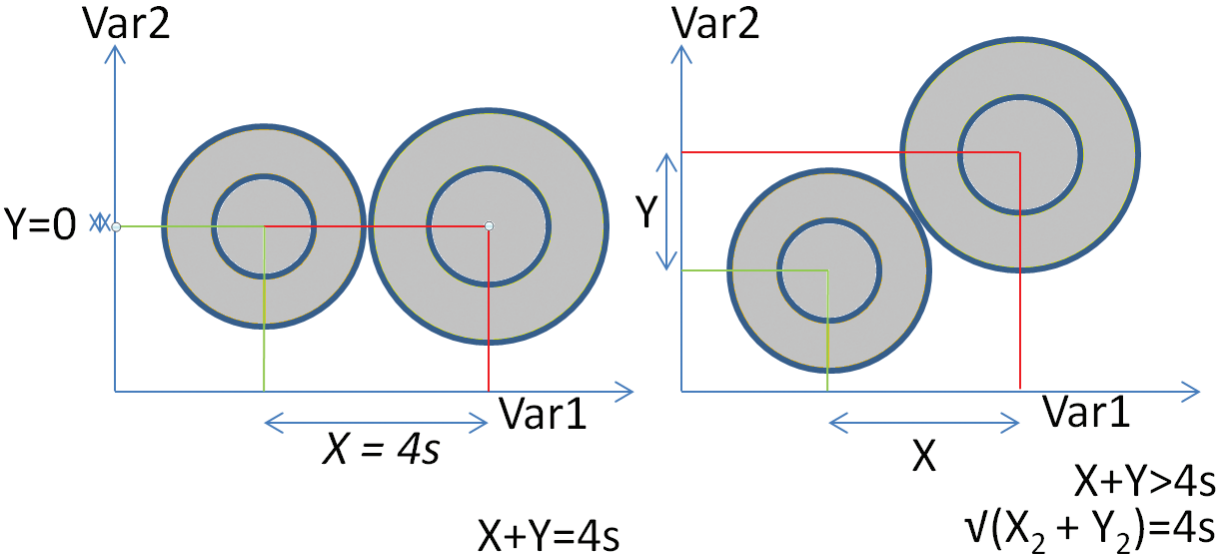
Justification for Euclidian summation, using a univariate/bivariate example.

However, this would be sub-optimal statistically. Applying such a formula would reflect the underlying conceptual approach of existing systems to rank allopatric populations (including Tobias et al. 2010), which afford weighting to distances in multiple variables by simple addition. However, in bivariate or multivariate space, a distance based on simple addition of mean differences is overly liberal. In Figure 8, the two circles represent two populations of different standard deviation, with each ring representing one controlled unpooled effect size from the centroid for a relevant variable and each population just being diagnosable from the other. In univariate space, when Var2 does not vary, then the difference between the two populations in Var1 (*x*-axis) is equal to the total variation. However, simple summation of the differentiation between Var1 and Var2 over-estimates the actual distance between the centroids in bivariate space. With two data variables, the distance between the data set (*a*_1_, *a*_2_) and (*b*_1_, *b*_2_) is not | (*a*_1_-*b*_1_) | + | (*a*_2_-*b*_2_) | but √[(*a*_1_-*b*_1_)^2^ + (*a*_2_-*b*_2_)^2^] (Fig. 8). When analyzing the multi-dimensional points as follows:

(*a*_1_, *a*_2_, *a*_3_, *a*_4_ …. *a*_n_) and (*b*_1_, *b*_2_, *b*_3_, *b*_4_ … *b*_n_),

then Pythagorian principles result in the following calculation of distance between points *a* and *b* in multi-dimensional space:

√[(*a*_1_–*b*_1_)^2^ + (*a*_2_–*b*_2_)^2^ + (*a*_3_–*b*_3_)^2^ … + (*a*_n_–*b*_n_)^2^]

And this can be simplified to:

√(∑ (*a*_n_–*b*_n_)^2^)

This approach cannot perfectly be applied to a series of effect size measures based on multiple pairwise comparisons, in that such data are not necessarily linked to one another as a set of corresponding coordinates. However, assuming that the variables studied are independent, it is valid to measure distance this way. Independence of variables can be verified through correlation tests and promoted in variable selection by seeking to capture the maximum possible observed variation efficiently.

Each controlled unpooled effect size (that has not been eliminated to zero using the statistical significance filter) can be considered to represent the equivalent of a distance |*a_n_*-*b_n_*|. The distance in multi-dimensional space between the two populations is better approximated than through simple addition by taking the square of each controlled unpooled effect size (which has not been excluded due to non-significance), adding those up, and then calculating the square root of the sum of all of them.

### A new universal formula to determine taxonomic rank of allopatric populations using continuous variables

In studies using continuous variables, allopatric populations should be ranked as species if they show equal to or greater variation than that shown between closely related sympatric species (Helbig et al. 2002). This can be measured by carrying out pairwise comparisons to calculate effect sizes for all variables under study, controlling all these effect sizes for sample size using *t*-distributions, excluding all statistically insignificant outcomes and applying Euclidian summation.

*Viz*, an allopatric population will be a candidate for species rank if:

√(∑ [*p*<0.05/*n*_v_ → |(*x̄*_1_–*x̄*_2_)| / ¼[*s*_1_(*t*_1 @ 97.5%_) + *s*_2_(*t*_2 @ 97.5%_)]] ^2^)

≤

√(∑ [*p*<0.05/*n*_v_ → |(*x̄*_3_–*x̄*_4_)| / ¼[*s*_3_(*t*_3 @ 97.5%_) + *s*_4_(*t*_4 @ 97.5%_)]]^2^)

Where:

Species 1 and Species 2 are two sympatric species that are closely related to one another (preferably known to be sisters) and which are related to Population 3 and Population 4.

Population 3 and Population 4 are two allopatric populations whose rank is being determined.

*p*: the probability using Welch’s unequal variance *t*-test (or other similar technique for non-normally distributed data), as set out under Level 1 in Methods that the means of the populations differ.

*n_v_*: the number of continuous variables of a particular “family” considered in the study, so as to apply a Bonferroni correction.

1, 2, 3, and 4 refer to relevant data for Species 1, Species 2, Population 3 and Population 4 respectively.

*x̄*_1_, *x̄*_2_, *x̄*_3_, and *x̄*_4_ are the sample means of a relevant data set for a particular variable for Species 1, Species 2, Population 3 and Population 4, respectively.

*s*_1,_ *s*_2,_ *s*_3,_ and *s*_4_ are the standard deviations for a relevant data set for a particular variable for Species 1, Species 2, Population 3 and Population 4, respectively.

*t* refers to the *t*-value (based on *t*-distribution) using a one-sided confidence interval at the percentage specified for the relevant population and variable, with *t*_1,_ *t*_2,_ *t*_3_ and *t*_4,_ referring to such value for Species 1, Species 2, Population 3 and Population 4 respectively.

Because this formula is not simple to calculate, a spreadsheet is being published alongside this paper on the author’s researchgate.net site, which facilitates rapid calculations.

In some ways, when one looks carefully at the outcomes of different statistical tests undertaken here, the formula is a statement of the obvious. The statistics underlying it are basic. It merely relies upon the good aspects of long-established statistical methods for comparing continuous variables of previous authors (e.g., Hubbs and Perlmutter 1942, Amadon 1949, Isler et al. 1998, Tobias et al. 2010) and stands on their shoulders considerably. However, no one has to my knowledge or to date (at least in ornithology) proposed such a universal test of species rank of allopatric populations based on continuous variables into a single statistical framework.

As regards Isler et al. (1998)’s contributions, this formula borrows from their concept in using controlled unpooled effect size measures as its basis. However, their approach is genericized in a way potentially applicable to all taxonomic groups, given that different groups can show differing levels of intra-species variation when subjected to universal scoring systems (Donegan et al. 2015). Isler et al. (1998)’s additional test of non-overlap is discarded but a different gateway test, namely statistical significance, is added. Finally, effect sizes are applied in such a way as to take into account all validly measured variation, not just those variables which are diagnosable. This test also borrows from certain aspects of the Tobias et al. (2010) species-scoring technique – attributing value to a broader range of different effect sizes, including those below diagnosability, but unlike those authors discarding statistically insignificant data, using unpooled standard deviations and controlling better for sample size. Contrary to all previous models, a proportionate score based on a sliding scale is attributed, depending on how large the measured differences are. The test also has further benefits which are not delivered under any other systems to evaluate differentiation and taxonomic rank. First, it eliminates the possibility of non-statistically significant results affecting taxonomy at all. Secondly, it eliminates “hard cut-offs” entirely, other than in respect of the final determination of species rank. Thirdly, it is extensible. A study of two variables or of 100 variables can be applied equally into this framework. Fourthly, it is taxonomically neutral and can in principle be applied to any genus, family, order or class of any organism when continuous variables are studied, or indeed for any kind of statistical study using continuous variables in which diagnosability or comparability of differences is tested.

Some important recommendations should be borne in mind when using this method, in addition to those set out above under “Steps”:

(i) *Continuous versus non-continuous variables*: Some issues arose in the case studies here due to data gaps. In the studies of *Sirystes* and *Basileuterus*, non-homologous vocalizations were not compared with one another. There, populations with different measures for the same sorts of variables are recovered as more differentiated than those populations whose variables cannot validly be compared at all. Diagnosability based on the comparison of non-homologous vocalizations can be important, but it can also have pitfalls (notably, Chaves et al. 2000 claimed discrete variation in calls to claim sufficient differentiation under the Isler et al. (1998) model to support a split in antbirds, but homologous vocalizations existed that were ignored). Where populations differ principally by non-continuous rather than continuous variables and sample sizes are sufficient (Walsh 2000), then the scoring system of Tobias et al. (2010) (as modified here) is better applied, since that incorporates the study of both kinds of variables. In general, where populations are so different that variables cannot effectively or meaningfully be compared at all using continuous data, then this is likely of itself to be indicative of species rank. See further paragraph (iv) below.

(ii) *Sample sizes*: If either Species 1 or Species 2 have very low sample sizes, then: (i) for many variables under study, data may not meet the threshold test of statistical significance; and (ii) those which do could be affected by low standard deviations and inflated effect sizes caused by clustering. These issues apply in reverse where Population 3 or Population 4 suffers from such constraints. Although the test above is in principle sample size-neutral, caution should be exercised in interpreting results based on smaller samples (see *Myrmeciza* biometrics discussion below and Table 24). That said, small sample sizes are often an inescapable fact and, if this is the case, then we should feel comfortable about applying statistical methods such as these (which seek to address sample sizes to a particular confidence interval) and acting on the basis of their outcomes. We should also be enthusiastic about revising taxonomies and conclusions when further data implies a need to do so, rather than affording undue weight to *status quo* taxonomies or to older studies, which are usually based on even fewer data or even lower sample sizes.

(iii) *Scale factoring and manipulation through overloading*: Where there are 15 vocal measures and 5 biometric measures, it should be considered whether to weight scoring on a 50:50 basis, following Tobias et al. (2010)’s recommendations of equal weighting for different sources of data. There is a potential risk of misuse linked to scale factors. For example, if tail length varies between Population 3 and Population 4 but is equal between Species 1 and Species 2, it would seem inappropriate to increase the impact of this observation though ten separate tail feather measurements. With bill length, similarly, one could measure separately from the skull, nostril, and feathering to create three variables out of one; or biometric weightings could be tripled in impact with male, female and combined data or duplicated by using both museum specimen and live specimen data separately. In some cases, where all the wing feathers are also all measured, then measuring all the tail feathers might be appropriate, but such variables may then exceed the number of vocal variables in a study and require scale factoring. In some species, male and female songs are often very similar to one another; female songs showing similar patterns of between-population differentiation to male songs may be best excluded to avoid bias and doubling-up of scores. In some groups, biometrics may be more likely to be informative to taxonomy than voice or *vice versa*. Based on the case studies included here, which are largely of forest species where vocal characters are important, I will suggest that scale factoring is best addressed simply by an honest attempt to encapsulate the maximum possible extent of observed differences through the smallest possible number variables, with no more, even if (as in many studies here) this means giving equal weightings to tens of vocal measures and only five biometric measures. However, this suggestion should not discourage more in-depth and detailed studies involving weighting to avoid particular components becoming dominant. Isler et al. (1998) applied correlation tests to eliminate related variables, which should also be considered to improve the robustness of variable sets and is effectively a form of weighting. Remsen (2016) suggested studying different families for taxonomically informative characters, which could easily be incorporated into this framework.

(iv) *Not going over the top*: The formula presented here is proposed for usage in more difficult, borderline, or complicated cases. Where simpler studies can show allopatric populations or newly discovered populations to be very different indeed from one another, then there should be no need for a litany of statistical analyses to be undertaken. It should be appropriate in some cases simply for an author to publish photographs of specimens or sonograms or a brief subjective text to describe the differences observed. A good example of a situation in this category would be the allopatric Western and Eastern Woodhaunters *Automolus virgatus* and *A. subulatus*, whose vocalizations resemble one another not one iota (Ridgely and Greenfield 2001, Donegan et al. 2011) and show no mutual playback response (Freeman and Montgomery 2017), but whose split has not been universally accepted to date. Notably, Remsen et al. (2018) rejected this, one committee member considering that “there is value in requiring some minimum standards of published data for making taxonomic changes” and in a further ongoing attempt at promoting this change, another committee member has proposed rejection “out of principle”. Such approaches waste limited human taxonomic resources on simple situations and reinforce irrational taxonomies.

(v) *Possible usage of controlled pooled effect size*. The formula above does not use pooled standard deviations and so makes no assumptions about the comparability of the variances of the different populations under study. As discussed above, there may be use cases for controlled pooled effect size, especially as a hedge for small sample sizes, but this should be applied only with caution. In any cases where assumptions of equal SD may be made among all four populations 1, 2, 3 and 4, then a more complicated formula using controlled pooled standard deviations might be used instead (see Materials and methods for details of equations that may be substituted in).

### Example of using the test: *Myrmeciza* antbirds

*Myrmeciza* was chosen here as an example because the recommendations of the relevant paper (Donegan 2012) have been accepted by all relevant authorities (Remsen et al. 2018, Gill and Donsker 2018, Del Hoyo and Collar 2016) and are justified based on both the Isler et al. (1998) and Tobias et al. (2010) models. Moreover, the species rank candidates (Blue-lored Antbird *M. immaculata* versus Zeledon’s Antbird *M. zeledoni)* are sisters with respect to one another, as are the comparator sympatric pair (Goeldi’s Antbird *M. goeldii* versus White-shouldered Antbird *M. melanoceps*) (Isler et al. 2013). Vocal data is considered here primarily, since only two specimens of *M. goeldii* were found in the study, resulting in no statistical significance being recovered in any biometric comparisons and scores of 0 across the board. A simplistic comparison is first shown, of the Central Andes population *M. i. concepcion* versus the proximate Chocó population of *M. z. macrorhyncha*. In reality, the relevant allopatric species both each comprise two allopatric subspecies. Measures for each of the vocal variables in question can be inspected in Donegan (2012). Bonferroni correction at *p*<0.05/26 vocal variables produces *p*<0.0019 for voice.

Table 22 includes a work-through of the calculation under the new methodology proposed here. Scores, of *immaculata/zeledoni*: 13.75 > 7.13: *melanoceps/goeldii*, imply that differences in voice between the allopatric pair are greater than those between related sympatric pair. The requirement of the new formula is satisfied and *zeledoni* and *immaculata*, which were treated as subspecies under traditional taxonomies, are therefore valid species with respect to one another.

**Table 22.** Worked-through example of the new formula proposed herein, assessing the rank of two allopatric *Myrmeciza* antbirds (*M. immaculata* vs *M. zeledoni*) by comparison to a pair of sympatric sister taxa in the same genus (*M. goeldii* vs. *M. melanoceps*), using vocal data only.

Variable	*M. goeldii* vs. *M. melanoceps*			*M. immaculata* vs. *M. zeledoni*		
	Controlled unpooled effect size	*p* value	Score	Controlled unpooled effect size	*p* value	Score
**Male song**						
No. of notes	1.41	5.2 × 10^-29^	1.41	4.54	6.92 × 10^-33^	4.54
Song length	0.37	0.0015	0.37	0.71	0.0022	0
Song speed	2.05	3.9 × 10^-49^	2.05	7.65	1.03 × 10^-89^	7.65
Max. acoustic frequency second note	1.03	2.7 × 10^-16^	1.03	3.30	7.20 × 10^-18^	3.30
Max. acoustic frequency of last note	1.31	3.9 × 10^-23^	1.31	2.88	2.03 × 10^-15^	2.88
Change in acoustic frequency	0.52	3.7 × 10^-5^	0.52	1.36	2.62 × 10^-8^	1.36
Position of peak of frequency	4.75	4.5 × 10^-74^	4.75	0.08	0.76	0
Position of trough in frequency	3.83	9.5 × 10^-55^	3.83	0.03	0.91	0
**Single note call**						
Call length	0.91	0.00270	0	0.99	0.040	0
Maximum acoustic frequency	0.99	0.00103	0.99	0.37	0.16	0
**Multi-note call**						
No. of notes	0.11	0.770	0	0.01	0.99	0
Song length	0.06	0.880	0	0.22	0.57	0
Song speed	0.21	0.494	0	0.21	0.56	0
Max. acoustic frequency	0.31	0.371	0	1.71	0.00049	1.71
Min. acoustic frequency	0.19	0.529	0	2.29	0.00013	2.29
Change in acoustic frequency	0.24	0.447	0	0.45	0.25	0
Position of peak of frequency	0.49	0.396	0	0.65	0.12	0
Position of trough in frequency	0.02	0.946	0	0.12	0.74	0
**Female song**						
No. of notes	0.78	0.0073	0	5.52	2.41 × 10^-15^	5.52
Song length	0.60	0.034	0	1.00	0.023	0
Song speed	1.18	7.07 × 10^-5^	1.18	7.00	1.50 × 10^-19^	7.00
Max. acoustic frequency second note	0.61	0.031	0	1.62	0.0034	0
Max. acoustic frequency of last note	1.14	0.00020	1.14	2.61	3.86 × 10^-7^	2.61
Change in acoustic frequency	0.23	0.40	0	0.71	0.18	0
Position of peak of frequency	0.04	0.89	0	0.96	0.11	0
Position of trough in frequency	0.27	0.33	0	0.47	0.41	0
**Euclidian distance (square root of sum of the squares)**			**7.09** *(7.14 using data to more s.f.)*			**13.95** *(13.75 using data to more s.f.)*

Table 23 shows vocal scores for cross-comparisons of the entire study group in *Myrmeciza*. The two scores in italics are for those achieving only subspecies rank under this system, i.e., those populations failing to attain the 7.14 suggested benchmark for species rank in this genus. Other allopatric populations concerned have all speciated with respect to one another and were previously ranked in separate species under traditional taxonomies. Sooty Antbird *Myrmeciza fortis* is also sympatric with respect to both *M. goeldii* and *M. melanoceps*.

**Table 23.** Full scores across the *Myrmeciza* data set for vocal data only. Bold denotes a sympatric pair of sister taxa. Bold italics denote other sympatric pairs. Denote pairs (with asterisk) which are subspecies based on overall scoring and discrete characters (see also Table 24). All other comparisons are between allopatric populations ranked with respect to one another as species.

	*M. i. concepcion*	*M. z. macrorhyncha*	*M. z. zeledoni*	*M. fortis*	*M. goeldii*	*M. melanoceps*
*M. i. immaculata*	3.56*	12.22	11.23	28.00	18.15	14.60
*M. i. concepcion*		13.75	15.64	19.51	21.49	15.50
*M. z. macrorhyncha*			4.08*	23.74	21.13	17.85
*M. z. zeledoni*				28.45	28.15	24.39
*M. fortis*					***22.81***	***12.73***
*M. goeldii*						**7.14**

Biometric scores are shown in Table 24. Even if a 3.52 uplift could be applied to biometric scores for the sympatric pair, which would raise the overall benchmark to 10.66, *M. zeledoni* and *M. immaculata* still meet the required benchmark for species with respect to one another.

**Table 24.** Scores across the *Myrmeciza* data set for biometrics. All *goeldii* scores (sample size *n*=2 specimens) were actually zero. Square bracketed figures showing alongside *M. goeldii* are based on controlled effect sizes without deleting insignificant data and are presented for reference only Bold denotes a sympatric pair of sister taxa. Bold italics denote other sympatric pairs. Denote subspecies (with asterisk) based on overall scoring (see also Table 23). All other comparisons are between allopatric populations ranked with respect to one another as species.

Taxon	*M. i. concepcion*	*M. z. macrorhyncha*	*M. z. zeledoni*	*M. fortis*	*M. goeldii*	*M. melanoceps*
*M. i. immaculata*	0*	3.02	1.72	3.35	[5.36]	5.96
*M. i. concepcion*		2.49	0	3.01	[5.13]	5.72
*M. z. macrorhyncha*			1.64*	2.22	[3.01]	5.07
*M. z. zeledoni*				2.54	[4.59]	5.35
*M. fortis*					[***3.47***]	***3.58***
*M. goeldii*						[**3.52**]

### What sorts of scores are good enough for assessing species and subspecies rank?

As above, although a universal *formula* is proposed here, no universal *score* is proposed here for ranking species, since the differentiation required to rank a species is likely to vary depending on the number of variables studied and by taxonomic group (Donegan and Avendaño 2008, Donegan et al. 2015). Simply, the score given to the allopatrics under study must exceed that of the related sympatrics.

There are however some parameters and examples available from the case studies (Table 25). The range of scores here for sympatrics may or may not be typical. Some presently recognized allopatric species scored less than these scores in some studies. A score of 4 under this system should be regarded as a *very bare minimum* for any proposal to rank a species based solely on continuous data. At that point, the two populations are differentiated in multi-dimensional space to 95% confidence. However, the actual value (being greater than 4) that it is necessary so as to afford species rank will depend on the data set and intra-specific variation in the group under study.

**Table 25.** Scores of examples from the data set which are both (i) sister species (or relevant sympatric subspecies of sister species) as shown by molecular studies; and (ii) sympatric, to show ranges of scores. Also presented are examples of scores passing other authors’ species tests. Note the Isler et al. (1998) score is likely an underestimate these since it does not take into account any non-diagnosable but significant other variation. Note that Tobias et al. (2010) allows only a maximum of four continuous variables (2 biometrics, 2 vocal) to be counted, so should be similarly interpreted as conservative scores.

Sympatric pair or proposed score for species rank	Type of data	Score
*Myrmeciza goeldii* vs *Myrmeciza melanoceps*	Voice	7.14
*Scytalopus griseicollis griseicollis* vs. *Scytalopus spillmanni* undescribed East Andes population	Voice + biometrics = total	9.16 + 0 = 9.16
*Scytalopus griseicollis gilesi* vs. *Scytalopus spillmanni* undescribed East Andes population	Voice + biometrics = total	10.59 + 0 = 10.59
*Scytalopus griseicollis morenoi* vs. *Scytalopus spillmanni* undescribed East Andes population	Voice + biometrics = total	8.79 + 0 = 8.79
*Grallaricula ferrugineipectus* Venezuela vs *G. nana nanitaea* Merida Andes	Voice + biometrics = total	7.90 + 8.01 = 15.91
***Average ± s.d. (min.–max.) (n=sample number)***		**10.32 ± 3.36 (7.14**–**15.91) (*n*=5)**
Basis for Isler et al. (1998) model	Voice: diagnosability of three characters = 3 × 4 SD	6.92
A basis for Tobias et al. (2010) score of 7 for species rank	Voice or biometrics: 1 × 10 SD (score 4), 1 × 5 SD (score 3)	11.18
A basis for Tobias et al. (2010) score of 7 for species rank	Voice and biometrics: 1 × 10 SD (score 4), 1 × 2 SD (score 2), 1 x 0.2 SD (score 1)	10.20
A basis for Tobias et al. (2010) score of 7 for species rank	Voice and biometrics: 1 × 10 SD (score 4), 3 × 0.2 SD (score 1 each)	10.01
A basis for Tobias et al. (2010) score of 7 for species rank	Voice and biometrics: 2 × 5 SD (score 3 each), 1 × 0.2 SD (score 1)	7.07
A basis for Tobias et al. (2010) score of 7 for species rank	Voice and biometrics: 1 × 5 SD (score 3), 2 × 2 SD (score 2 each)	5.74
A basis for Tobias et al. (2010) score of 7 for species rank	Voice and biometrics: 1 × 5 SD (score 3), 1 × 2 SD (score 2), 2 × 0.2 SD (score 1 each)	5.39
A basis for Tobias et al. (2010) score of 7 for species rank	Voice and biometrics: 3 × 2 SD (2 each), 1 × 0.2 SD (1 each)	3.47

As regards subspecies or PSC species, any score of 4 or more (i.e., allowing full diagnosis in multidimensional space) would be a supportable benchmark. There may however be cases of valid subspecies which achieve lower scores than this, such as in the *Adelomyia melanogenys* study where the pair discussed above scored only 2.10 for voice and 0 for biometrics, based on a fairly exhaustive attempt at measuring biometric and vocal variables. However, this is probably an exceptionally low-scoring example.

Among the un-named populations in the study group, only the “Apurímac south” population of *Basileuterus tristriatus* in Peru was recovered as diagnosable versus all proximate subspecies (6.33 versus Marañon to Apurímac population, 14.79 versus Bolivia) and therefore requires formal description. Other notable unnamed populations include the Tamá population of *Grallaricula nana* (scores 3.32 versus Mérida) and the West Andes populations of the same species (scores 2.86 against Central Andes). The two new *Grallaricula* taxa described in Donegan (2008) each scored over 4 compared to proximate populations. A noteworthy split proposed by Donegan (2008) but rejected by all relevant learned taxonomic committees (Remsen et al. 2018, Gill and Donsker 2018, Del Hoyo and Collar 2016) is that of *Grallaricula nana kukenamensis*, which scored 6.24–11.19 on biometric data alone compared to all other populations in the *nana* group with which it is purportedly lumped – although only a single tentative sound recording remains available. Such biometric differentiation exceeds that of almost all sympatric pairs studied here (including many that are not sister taxa). Taking into account plumage differences, it also exceeds the scoring benchmark of Tobias et al. (2010) for species rank.

Probably the most difficult taxonomic decision in this series of papers was that of how to rank *Scytalopus rodriguezi*, whose allopatric subspecies scored 5.40 for biometrics and 5.01 for voice, total 10.41 and so was more diagnosable than some sympatric tapaculos. A large component of this score (compared to sympatric pairs) was in biometrics and the two populations were found to respond to one another’s playback. In borderline cases such as this, where different kinds of variables differ between the sympatric pair and allopatric pair, then scale factoring may be appropriate. Voice is a very important character for tapaculos and in this case, the vocal score, whilst showing full diagnosis, did not attain the differentiation shown between known sympatric comparators.

The Tobias et al. (2010) system produces some scores which are consistent with the differences between sympatric species studied here. However, species can be justified under that scoring system based on wildly differing measured variation (Table 25). The quest for a universal scoring system requires a more exacting basis for calculations in order to produce the rational taxonomy that it seeks. It was most surprising in this study that the measured differentiation here between sympatric *Scytalopus* exceeded that between sympatric *Myrmeciza*. In contrast, as mentioned in the introduction, Donegan and Avendaño (2008) found the same sympatric tapaculo pairs to differ to a lesser extent than sympatric antbirds under Isler et al. (1998)’s framework. The present study shows that when below-diagnosis differentiation in other characters is taken into account (see Figure 5), sympatric tapaculos are much closer in their vocal differentiation to sympatric antbirds.

The method proposed here involves no universal score for species rank. However, it would still be interesting to see how other pairwise situations involving sympatric sister species measure up under this system, and then possibly to revisit the philosophy underlying Tobias et al. (2010)’s methods accordingly.
